# An Atlas of Promoter Chromatin Modifications and HiChIP Regulatory Interactions in Human Subcutaneous Adipose-Derived Stem Cells

**DOI:** 10.3390/ijms25010437

**Published:** 2023-12-28

**Authors:** Laszlo Halasz, Adeline Divoux, Katalin Sandor, Edina Erdos, Bence Daniel, Steven R. Smith, Timothy F. Osborne

**Affiliations:** 1 Division of Diabetes Endocrinology and Metabolism, Departments of Medicine, Biological Chemistry and Pediatrics, Johns Hopkins University School of Medicine, Institute for Fundamental Biomedical Research, Johns Hopkins All Children’s Hospital, St. Petersburg, FL 33701, USAtosborn9@jh.edu (T.F.O.); 2 Translational Research Institute, AdventHealth, Orlando, FL 32804, USA; steven.r.smith@adventhealth.com

**Keywords:** adipose tissue, adipose-derived stem cell, transcriptome, chromatin, 3D organization, epigenome

## Abstract

The genome of human adipose-derived stem cells (ADSCs) from abdominal and gluteofemoral adipose tissue depots are maintained in depot-specific stable epigenetic conformations that influence cell-autonomous gene expression patterns and drive unique depot-specific functions. The traditional approach to explore tissue-specific transcriptional regulation has been to correlate differential gene expression to the nearest-neighbor linear-distance regulatory region defined by associated chromatin features including open chromatin status, histone modifications, and DNA methylation. This has provided important information; nonetheless, the approach is limited because of the known organization of eukaryotic chromatin into a topologically constrained three-dimensional network. This network positions distal regulatory elements in spatial proximity with gene promoters which are not predictable based on linear genomic distance. In this work, we capture long-range chromatin interactions using HiChIP to identify remote genomic regions that influence the differential regulation of depot-specific genes in ADSCs isolated from different adipose depots. By integrating these data with RNA-seq results and histone modifications identified by ChIP-seq, we uncovered distal regulatory elements that influence depot-specific gene expression in ADSCs. Interestingly, a subset of the HiChIP-defined chromatin loops also provide previously unknown connections between waist-to-hip ratio GWAS variants with genes that are known to significantly influence ADSC differentiation and adipocyte function.

## 1. Introduction

The distribution of adipose tissue throughout the body plays a significant role in predicting the health status of overweight and obese people independent of body mass index (BMI) [1,2]. Excess accumulation of fat in the upper body (apple-shaped) is positively correlated with higher HbA1c, circulating triglycerides (TG), and adverse serum lipid profiles [3]. In contrast, excess accumulation of fat in the lower body (pear-shaped), such as in the gluteofemoral (GF) depot, is negatively correlated with the same metabolic disease markers [4].

A recent theory to explain the effect of differential fat accumulation on metabolic health posits that the lower body adipose tissue serves as a sink for “healthy” lipid deposition and limits fat accumulation in the upper body, notably in visceral adipose tissue, the latter of which is associated with chronic inflammation and insulin resistance [5,6]. Although it is well established that growth hormone, cortisol, and sex steroids influence fat distribution [7,8,9], the underlying mechanism for why people develop the apple vs. pear body shape is still not completely understood. At the cellular level, besides the obvious role of mature adipocytes to sequester lipids, there is a likely contribution from the precursor cells or adipose-derived stem cells (ADSCs) which have the capacity to differentiate into mature adipocytes to store excess lipid. In fact, previous studies have demonstrated that human primary subcutaneous abdominal (ABD) vs. GF-ADSCs, have differential adipogenic capacity in vitro [10]. In addition, there are distinct transcriptional signatures, chromatin marks, and DNA methylation patterns in ABD vs. GF adipose tissue [11,12,13] that are partially maintained in isolated ADSCs following culture in vitro [14,15]. Taken together, these observations provide evidence for an underlying epigenetic memory that contributes to the different patterns of gene-expression-driven phenotypes. Our working hypothesis is that these cell-autonomous epigenetic programs maintain unique ABD- and GF-AT characteristics in the cultured ADSCs and contribute to unique ABD and GF adipose tissue characteristics. These earlier studies integrated gene expression and individual epigenetic marks to help explain the sustained patterns of differential gene expression in ADSCs that were isolated from different subcutaneous adipose depots and cultured over several rounds of cell division. In the current work, we aim to extend these preliminary results and interrogate the epigenomic landscape of ABD and GF-ADSCs around the TSS of the differentially expressed genes between the two adipose tissue depots by using an extensive ChIP-seq analysis for multiple histone marks (H3K4me3, H3K4me2, H3K27me3, H3K9me3), CTCF, and RNA polymerase II along with ATAC-seq to probe chromatin openness.

In the earlier studies mentioned above, gene annotation of the regulatory regions was performed using a nearest-neighbor linear-distance approach which only showed a modest connection between differential chromatin features and differential gene expression [14,15]. We recognized this was an overly simplistic approach because chromatin is highly organized and condensed with DNA packaged in a highly ordered fashion with histones and other proteins into a complex three-dimensional network [16,17]. The resulting highly condensed chromatin serves to position distally located regulatory elements close to proximal gene promoters that would otherwise be located far away from each other based on a linear (2D) view of the genome. High-resolution three-dimensional methods including Hi-C and ChiAPET were developed that capture these long-range interactions after partial digestion of the DNA followed by ligation of closely juxtaposed ends that are brought into close proximity by looping out of the intervening DNA [18,19,20].

In the second part of the current work, we aimed to determine how the differential gene expression patterns in ABD and GF-ADSCs were significantly influenced by chromatin modifications and long-range chromatin interactions using the related HiChIP method which, when focused on H3K27ac, will identify loops that are anchored through an active transcriptional enhancer region [21]. We next integrated the HiChIP data set with our differentially expressed RNA-seq data set that compares gene expression patterns in ABD vs. GF-derived ADSCs. These data were then compiled into an atlas that combines the different chromatin marks and active enhancer connectome related to gene expression patterns for ABD vs. GF-ADSCs isolated from apple and pear-shaped women.

This in-depth analysis of the chromatin structure and organization of ABD and GF-ADSCs provides both an initial in-depth understanding of the intrinsic genomic regulatory features that influence the functionally distinct cellular phenotypes of ABD and GF subcutaneous adipose tissue depots, and a resource for the adipose tissue research community. We also demonstrate the value of this data set as a resource by cross-referencing this information with a data set of WHR-related SNPs; together, these investigations provide significant new information for how different adipose depots contribute to differential adipose patterning and metabolic disease risk in humans.

## 2. Results

**Figure 1:**
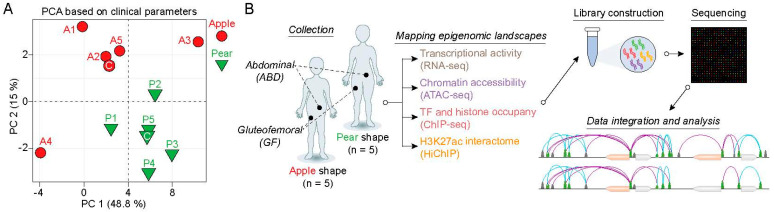
Sample acquisition and study design. (**A**) Principal component analysis (PCA) plot of the ten subjects used to isolate the ABD and GF-ADSCs based on clinical parameters. The first two principal components (PC) are plotted and colored according to body shape. PCA was performed using all clinical data collected during the clinical study. (**B**) Overview of the experimental workflow. Subcutaneous adipose tissue biopsies were performed on five apple-shaped and five pear-shaped subjects. From each biopsy, the stroma vascular fraction was isolated and the ADSCs were cultured in media supplemented with serum and growth factors. Cells were harvested, their chromatin was isolated and used for RNA isolation, ChIP, ATAC, and HiChIP assays, followed by sequencing.

To evaluate the cell-autonomous differences and explore the molecular regulation of gene expression between ABD and GF adipose tissue depots, we isolated adipose-derived stem cells (ADSCs) from paired ABD and GF adipose tissue originating from five apple and five pear-shaped women. Principal component analysis using all the clinical parameters (the most relevant are listed in Supplementary Table S1), showed that individuals within each group are highly similar and that the two groups are well separated from each other (Figure 1A). For this reason, we analyzed the apple and pear samples separately. The ADSCs were all cultured the same way and passaged the same number of times (±1) (see Section 4 for details) prior to harvest. The overall workflow of the study is described in Figure 1B. In summary, we performed ChIP-seq for histone marks, CTCF, and RNAPII along with ATAC-seq on freshly isolated chromatin. An additional aliquot of cells was frozen and used for RNA-seq analysis. We also performed H3K27Ac-enriched HiChIP to evaluate the 3D organization of the active enhancer connectome.

### 2.1. Transcriptomic Signatures of ABD and GF-Adipose-Derived Stem Cells

**Figure 2:**
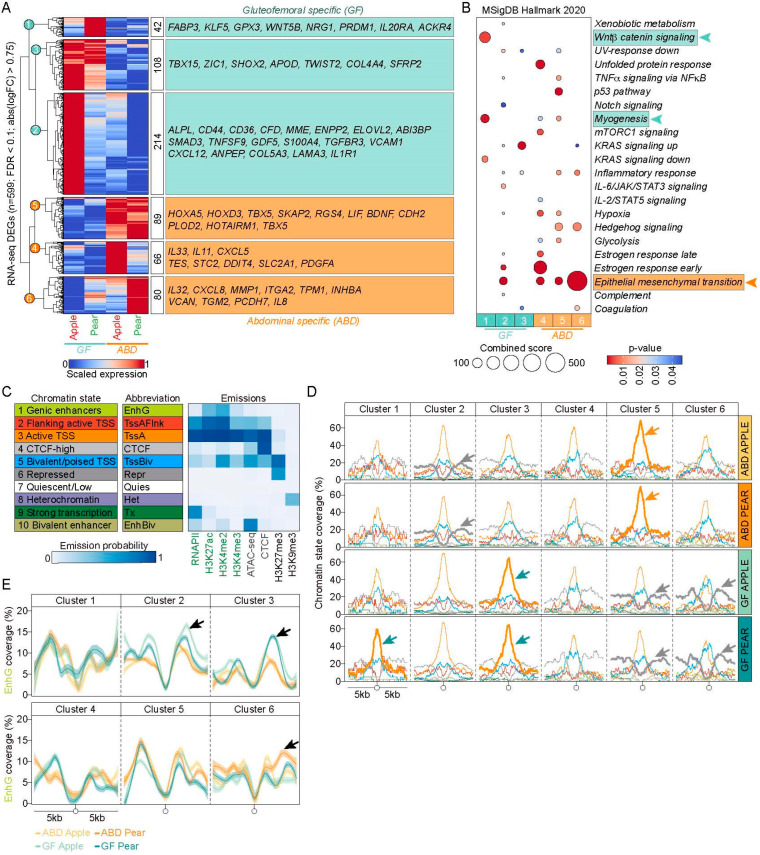
Depot-enriched gene expression and chromatin modification analysis of human ADSCs according to body shape. (**A**) Heat map showing the differentially expressed genes between ABD and GF-ADSCs in apple and pear-shaped subjects based on RNA-seq. The DEGs were grouped into clusters according to their level of expression in apple and pear samples. DESeq2 analysis, FDR < 0.01, FC > 0.75 Genes potentially involved in adipose tissue expansion are cited. (**B**) Dot plot showing the significant pathways of the DEGs in each cluster. Only the pathways with *p* < 0.05 are represented. (**C**) Legend showing the ChromHMM annotated states, with their emission values for individual chromatin marks. (**D**) Visualization of selected chromatin states (2, 3, 5, 6, 9, 10) around the TSS (±5 kbp) of DEGs per depot and body shape groups (rows) within gene clusters (columns). Arrows highlight when the states are visually different between ABD and GF. (**E**) Visualization of Genic enhancer chromatin state (state#1) around the TSS (±5 kbp) of DEGs per groups (colored) within gene clusters. Arrows highlight when the state is visually different between the ABD and GF samples.

We first identified the differentially expressed genes between ABD and GF-ADSCs using RNA-seq. Setting a cut-off of a 1.7-fold change and an FDR of 0.1, the RNA-seq analysis showed a total of 599 differentially expressed genes (DEGs) between the ABD and GF-ADSC samples, of which 364 exhibited GF-enriched features and 285 were ABD-enriched. This analysis revealed six clusters of DEGs (Figure 2A), stratified based on their level of expression in apple- and pear-shaped subjects.

Among the forty-two GF-enriched genes highly expressed in GF pear samples (Figure 2A, cluster 1), we found six pro-adipogenic marker genes (*GPX3*, *PRDM1*, *FABP3*, *KLF5*, *WNT5B*, and *NRG1* [22]) which would be consistent with GF-ADSCs in pear-shaped women having the capacity to differentiate more robustly compared to GF-ADSCs from apple-shaped women. Importantly, further analysis revealed that the most significantly enriched pathway in cluster 1 contained genes involved in Wnt–β-catenin signaling (Figure 2B), which is known to play a significant role in adipocyte differentiation [23]. More recently, an extensive analysis combining scRNA-seq combined with a xenograft mouse model as validation, showed that Wnt signaling preserves progenitor cell multipotency during adipose tissue development [24], which would be predicted to ensure a healthy pool of progenitor cells capable of differentiation to mature adipocytes.

Cluster 2 includes 214 GF-enriched genes highly expressed in apple-shaped samples (Figure 2A), several of which are important for lipid droplet formation and others that increase in expression during adipogenesis, such as *ALPL*, *CD44*, *CD36*, *CFD*, *MME*, *ENPP2*, *ELOVL2*, and *ABI3BP*. Cluster 2 also contains fibroblastic or fibrotic marker genes (*TGFBR3*, *LAMA3*, *TNFSF9*, *S100A4*, *VCAM1*, *CXCL12*, *ANPEP*, *COL5A*) not found in cluster 1. These genes might participate in the formation of collagen, which can form a scaffold that constrains adipocyte expansion due to mechanical stress in GF apple samples [25,26].

Cluster 3 includes 108 DEGs enriched in both apple and pear GF samples (Figure 2A). Some of these were previously identified as GF-enriched markers for whole adipose tissue (*TBX15*, *SHOX2*, and *SFRP2* [13,27]). Importantly, we also identified new depot-specific marker genes that are known to influence adipose tissue function (*ZIC1*, *TWIST2*, *COL4A4*, *APOD* [28,29,30,31]).

The 285 ABD-enriched genes were divided into three clusters of roughly equal size (clusters 4, 5, and 6—Figure 2A) based on their body shape expression pattern. Cluster 4 includes genes highly expressed in ABD apple samples but with low expression in GF pear samples. This cluster includes genes activated by hypoxia (*TES*, *STC2*, *DDIT4*, *SLC2A1*), cytokine/chemokine genes (*IL33*, *IL11*, *CXCL5*), and *PDGFA*, which is known to stimulate adipose progenitor proliferation and self-renewal but also is associated with increased adipose tissue fibrosis [32,33]. Cluster 4 also contains two other genes that may influence adipose tissue expansion: *BAMBI*, a gene known to regulate reactive oxygen levels [34] and *PAWR*, a suppressor of p53 [35].

Cluster 5 contains ABD-enriched genes highly expressed in both apples and pears, several of which have already been identified as ABD-enriched markers in whole adipose tissue (*HOXA* [27] and *HOXD* cluster genes, *TBX5*, and *HOTAIRM1* [36]) and others that have been previously associated with type 2 diabetes pathogenesis (*TBX5*, *PITX2*, *SKAP2* [37]) (Figure 2A). Genes known to limit adipose tissue expansion were also contained in cluster 5 (*LIF*, *PLOD2*, *BDNF*, and *CDKN2B* [38,39,40]).

Finally, cluster 6 includes genes highly expressed in ABD pear samples but with low expression in the other three samples, especially GF apple (Figure 2A). Interestingly, the pathway with the highest enrichment score in cluster 6 includes 80 genes related to epithelial–mesenchymal transition (EMT) (Figure 2B). Further analysis revealed these genes are also potential inhibitors of adipogenesis (*INHBA*, *ITGA2*, *IL32*, *OXTR*, *CXCL8*, *MMP1*, *TPM1*, *TFPI2*, *MYLK*, *VCAN*, *COL7A1*, *PMEPA1*, *TGM2*) [41] and this correlates well with our overall developmental/physiological hypothesis that there is reduced expansion of the ABD depot in pear subjects.

The MCODE analysis [42] revealed protein–protein interactions between the DEGs (Supplementary Figure S1). This analysis identified several proteins interacting with each other. Notably, FMN1 (up in GF depot) and EDN1 (up in ABD depot) were found at the center of two hubs, related to cell growth and inflammation, respectively. Several collagens were also interacting to a high degree. These proteins could be master regulators of the DEGs between ABD and GF-ADSCs and potential therapeutic targets to favorize lipid accumulation in the lower body adipose tissue rather than in the upper body depots.

### 2.2. Selective Epigenetic Hallmarks of Depot-Selective Transcription

The fact that several of the GF and ABD-ADSC-enriched genes were also enriched in the same samples from whole adipose tissue indicates the differential expression pattern is a stable feature that is likely maintained at least in part through depot-selective epigenetic regulation [14,15]. To evaluate genomic signatures that might contribute to the differential gene expression patterns highlighted above, we compared the chromatin features present in ABD vs. GF-ADSCs in the same samples used for the RNA-seq analysis. We performed ATAC-seq to evaluate chromatin openness and ChIP-seq targeting different histone marks (active chromatin: H3K27Ac—active enhancers/promoters, H3K4me2—active enhancers/promoters, H3K4me3—mainly active promoters; repressed chromatin: H3K27me3—facultative heterochromatin, H3K9me3—constitutive heterochromatin), CTCF (genome architectural protein), and the elongating form of RNAPII (phospho Ser 2 in CTD). Then, we integrated the chromatin modification data from all the samples to generate a combinatorial set of emission states using ChromHMM [43]. Emission parameters were learned de novo based on genome-wide recurrent combinations of the chromatin marks studies (see above) in ADSCs (ABD and GF combined). Importantly, each emission state was defined by a specific combination of chromatin features that may be associated with distinct biological expression patterns of their linked genes.

We distinguished 10 broad classes of chromatin emission states that are labeled according to their combined predicted influence on gene activity. These include “Genomic enhancers”, “Flanking Active TSS”, “Active TSS”, “CTCF-high”, “Bivalent/poised TSS”, “Repressed”, “Quiescent”, “Heterochromatin”, “Strong Transcription”, “Bivalent Enhancer” (Figure 2C), and they are independent of the tissue depot or body shape chromatin source. As a first step in understanding how these combined features influence gene activity, we displayed the average read density scores for the ten unique chromatin states around the transcription start site (TSS) of the DEGs in the two different depots (ABD and GF) and from the two different body shapes (apple and pear) in Figure 2D. Only three emission states were enriched at the TSS of differentially expressed genes. The first one “active TSS” (state 3, orange on the graphs in Figure 2D), contains all activate chromatin-associated histone marks along with CTCF, RNAPII, and the open chromatin signature (ATAC-seq peak) combined with very low levels of the repressive marks H3K27me3 and H3K9me3 (see legend Figure 2D). The active TSS state was enriched at the TSS of the GF-enriched genes belonging to clusters 1 and 3 in the GF samples (dark green arrows in Figure 2D) and at the TSS of the ABD-enriched genes belonging to cluster 5 in the ABD samples (dark orange arrows in Figure 2D).

The second enriched emission state corresponded to repressed regions (state 6, gray on the graphs in Figure 2D) which are characterized by an enrichment of the repressive H3K27me3 mark in the absence of the other features. The genes in clusters 5 and 6 are marked by a high level of the H3K27me3 repressive mark (grey arrows) at the TSS in the GF samples. This suggests that these genes are more highly expressed in ABD-ADSCs because their expression is repressed in the GF region. Taken together, these results support the concept that differential combinations of active and repressive chromatin marks at DEG’s TSSs contribute to depot-specific gene expression patterns in ABD and GF-ADSCs. We also found ‘bivalent domains’ of histone modifications (i.e., harboring both the repressive mark H3K27me3 and the activation-associated marks) near the TSS of genes with depot-specific expression (blue lane, Figure 2D).

To obtain a better assessment of the enhancer regions in the ABD vs. GF samples, we plotted only the “Genic enhancer” state (state 1 in Figure 2C) around the promoter of the DEGs. Figure 2E displays the average read density scores for this specific state around the TSS of the DEGs for the four different groups (ABD and GF, apple and pear subjects). The genes in clusters 2 and 3 (GF-enriched genes) are marked by an increase in enhancer marks in the GF samples whereas the genes in cluster 6 (ABD-enriched genes) are marked by an increase in enhancer marks in the ABD samples. These observations suggest an active role of enhancer genomic regions in depot-specific gene regulation in human ADSCs.

### 2.3. Alteration of Active and Repressive Epigenetic Marks Associated with Depot-Selective Gene Expression

**Figure 3:**
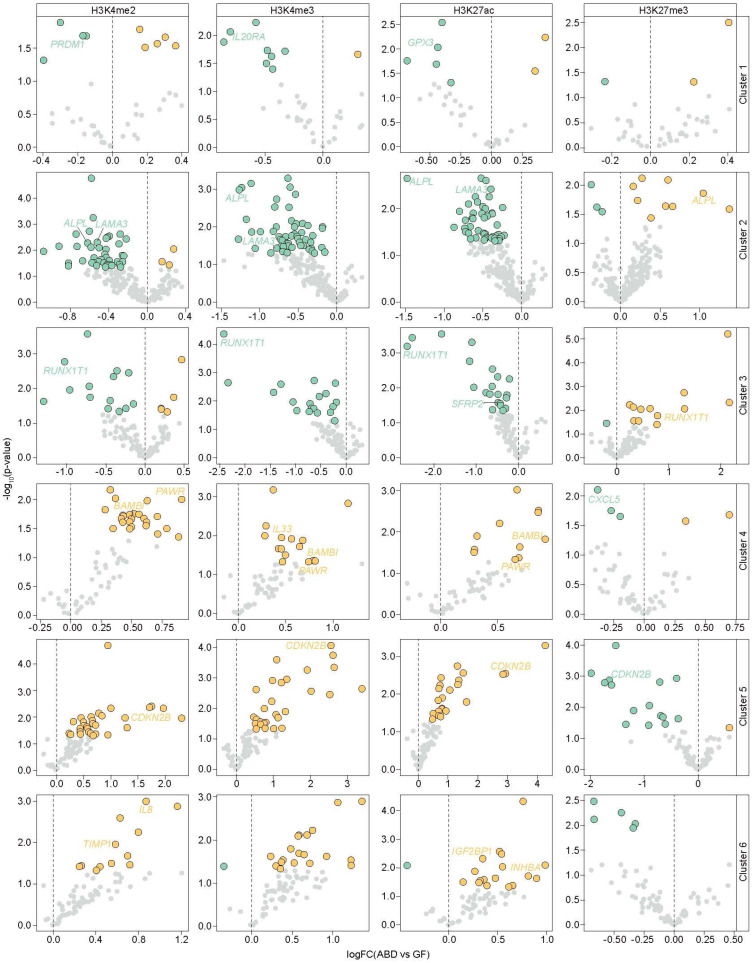
Association between depot-enriched expression and depot-enriched chromatin marks at the TSS (±2 kbp) in apple samples. Volcano plots show for each gene and each histone mark studied the average fold change of the ChIP-seq signal between ABD and GF-ADSCs at the TSS. Data are represented by cluster of DEGs (rows). Negative fold changes (green) indicate the ChIP-seq signal is significantly enriched in the GF samples, while positive fold changes (orange) indicate the ChIP-seq signal is significantly enriched in the ABD samples.

To evaluate the individual contributions of histone modifications in depot-selective gene expression, we separately analyzed the ChIP-seq marks within the TSS of the DEGs and compared the data between the ABD and GF-ADSCs. We focused on the individual histone marks that define the active TSS state (H3K4me3, H3K27Ac, and H3K4me2) and repressed state (H3K27me3), and calculated the differences in their respective ChIP-seq signals in ABD and GF-ADSCs around the TSS (±2 kbp) of the ABD and GF-DEGs. Those reaching statistical significance (*p* < 0.05) are colored (orange for ABD-enriched histone mark and green for GF-enriched histone mark) in the volcano plots of Figure 3 (apple subjects) and Supplementary Figure S2 (pear subjects). The full list of DEGs with the fold change for each histone mark is listed in Supplementary Table S2 for the apple subjects and Supplementary Table S3 for the pear subjects. For example, *HOXC11* and *TBX15* (GF-enriched genes, cluster 3) showed an enrichment of the active histone marks and a decrease of the repressive mark H3K27me3 in the ABD samples compared to the GF samples. This provides a more detailed evaluation of the association of positive and negative histone modifications with differential gene expression patterns in ADSCs and extends what has been described in other cancer cell model systems [44,45].

Our data also suggest that histone modifications affect expression of genes involved in adipogenesis such as *PRDM1*, *ALPL*, *RUNX1T1*, *SFRP2*, and *GPX3*. Importantly, the newly identified GF-enriched genes in our study, *ZIC1*, *GREM2*, and *IL20RA*, were also associated with GF-enriched differential patterns of histone modifications at their TSSs. Among the ABD-enriched genes within the highly active TSS emission state in ABD chromatin, we detected the key developmental genes *HOXA5*, *HOXD1*, *HOXD3*, *HOXD8*, and *TBX5* in cluster 5 (ABD-enriched genes in both body shape types). The differential chromatin marks were also associated with DEGs that are involved in adipose tissue expansion, such as *BAMBI*, *PAWR*, and *IL8* (clusters 4 and 6; ABD-enriched genes) along with other genes known to limit adipogenesis such as *INHBA* (cluster 6; ABD-enriched genes highly expressed in pear samples), *TIMP1*, and *CDKN2B* (cluster 5). Interestingly, two inflammatory genes (*CXCL5* and *IL33*) showed higher H3K27me3 levels around their TSSs in GF samples compared to their TSSs in ABD samples (cluster 4; ABD-enriched genes highly expressed in apple samples), suggesting that the expression of these two genes may be selectively repressed by H3K27me3 in GF-ADSCs.

### 2.4. HiChIP Regulatory Interactions in ABD and GF-ADSCs

In an earlier study comparing open chromatin regions identified by ATAC-seq with differentially expressed genes in freshly isolated adipocytes, we showed that only a small fraction of the body-shape-specific open chromatin regions were annotated to DEGs [46]. In this earlier study, we used linear distance as a guide suggesting that long-range genomic interactions mediated by chromatin looping are likely involved in the differential gene expression patterns. To determine how the differential gene expression patterns in ADSCs from different adipose depots may be influenced by long-range chromatin interactions, especially by enhancer genomic regions as suggested by our data in Figure 2E, we performed H3K27ac-targeted HiChIP on chromatin from ADSCs across the ten subjects and two adipose tissue depots.

**Figure 4:**
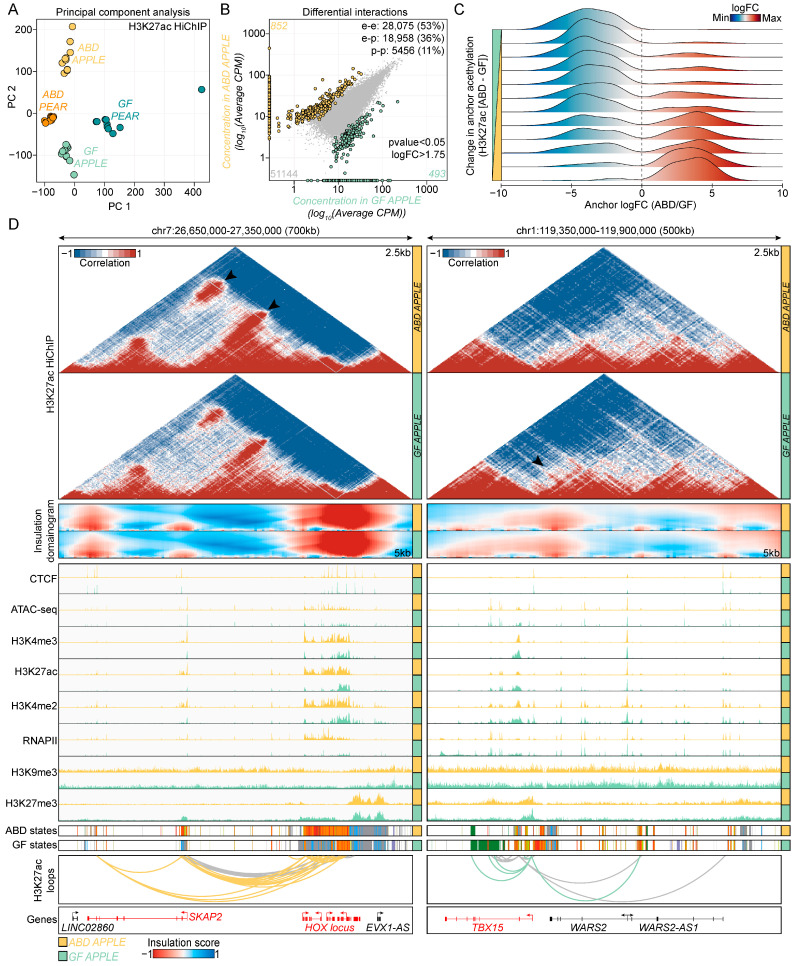
Mapping epigenomic landscapes in ABD and GF-ADSCs. (**A**) Principal component analysis (PCA) plot of normalized in-loop H3K27ac HiChIP read counts. The first two principal components (PC) are plotted and colored according to body shape. (**B**) Dot plot showing the correlation of read densities between ABD and GF-ADSCs in apple subjects. Differential loops are colored in yellow (ABD-enriched) and green (GF-enriched). The non-significant loops are represented in gray. *p*-value < 0.05 logFC > 1.75. (**C**) Density plot showing the correlation between differential looping (*x*-axis) and differential H3K27ac (*y*-axis) at loop anchors. The H3K27ac signal was binned into 12 groups based on the magnitude of difference in H3K27ac. Data were plotted for the apple subjects. Similar observations were made for the pear subjects. (**D**) Genome browser visualization of *SKAP2-HOX* locus (left) and *TBX15* locus (right) in ABD (yellow) and in GF (green) samples. Data were derived from apple subjects. Similar observations were made with data derived from pear subjects. From top to bottom: H3K27ac HiChIP interaction matrices, domainogram of insulation score, CTCF, ATAC-seq, H3K4me3, H3K27ac, H3K4me2, RNAPII, H3K9me3, H3K27me3, ChromHMM states, H3K27ac loops, and gene annotation. Color coding for ChromHMM plots is the same as Figure 2C.

We identified 52,489 and 52,615 loops in the apple and pear samples, respectively (hichipper, FDR < 0.01). Each sample had similar levels of high-quality uniquely mapped read pairs (Supplementary Figure S3A). Principal components analysis also showed that samples from each group clustered together, and their patterns were separated based on body shape and depot source (Figure 4A). Supplementary Figure S3B shows that the overall A/B compartment score distribution across all groups was identical for chromosome 7 and this was also evident on the whole genome level as shown by the saddle plots in Supplementary Figure S3C. The median loop length was 41 kb and as expected, the number of interactions decreased with increasing distance between loop anchors (Supplementary Figure S3D).

We then overlapped the anchors of the HiChIP loops with gene promoters and enhancers and the resulting loop sets were binned into three different categories: enhancer–promoter loops (18,958 in apples and 19,011 in pears), enhancer–enhancer loops (28,075 in apples and 28,138 in pears), and promoter–promoter loops (5456 in apples and 5466 in pears).

To identify differential loops between the ABD and GF-ADSC samples, we ran diffloop analysis separately for the apple and pear groups. This revealed 852 ABD-enriched loops and 493 GF-enriched loops in the apple samples with a *p* < 0.05 and fold change of 1.75 between the groups (orange and green symbols in Figure 4B). The number of depot-enriched loops was lower for the pear groups (304 ABD-enriched and 238 GF-enriched, Supplementary Figure S3E). To validate the depot-specific regulatory loops identified by HiChIP, we overlapped the loop anchors with the read density from the independently performed H3K27ac ChIP-seq analysis on the same set of samples from Figure 2 and Figure 3. The fold change between the ABD and GF HiChIP reads at the loop anchors highly correlated with the fold change between the ABD and GF H3K27ac signal detected by ChIP-seq at the same loop anchors. This correlation is consistent with the HiChIP pipeline used in our study, accurately identifying authentic depot-enriched loops.

This comparison resulted in a list of high-confidence H3K27ac loops in the ABD and GF-ADSC samples, including promoter and enhancer interactions that we analyzed further below. At 2.5 kb resolution, the H3K27ac HiChIP maps revealed depot-specific promoter–enhancer interactions at the promoter of HOXA genes in the ABD sample which are known ABD-enriched genes [27] (Figure 4D, left) and at the promoter of the TBX15 gene in the GF samples which is a known GF-enriched gene (Figure 4D, right). Additionally, TBX15 expression correlates with WHR and there is some evidence that it is a master transcriptional regulator in adipose tissue [47]. The genomic regions that were enriched in loops (HiChIP results) also were highly enriched for the CTCF ChIP-seq peaks that were identified in the CTCF ChIP-seq data used in the ChromHMM analysis in Figure 2C. These sites co-mapped with genomic regions with a high insulation score, consistent with CTCF-associated looping organizing the 3D genomic architecture to regulate gene expression. There was also strong enrichment for H3K27ac binding along with higher RNAPII and other marks associated with gene activation in ABD chromatin at the HOXA locus (Figure 4D, left IGV snapshots and ChromHMM). Similarly, the GF-enriched TBX15 HiChIP loops were associated with more robust peaks for active histone marks and RNAPII in the GF sample (Figure 4D, right IGV snapshots and ChromHMM).

### 2.5. Loop Anchors Harbor DEGs and SNPs That Are Associated with Waist–Hip Ratio in Humans

To further define regulatory regions that might influence differential gene expression between ABD and GF-ADSCs, we first identified the HiChIP loop anchors that were linked to DEGs. In apples, this revealed 323 loops in the genomic regions of the DEGs between the ABD and GF samples, and these loops mapped to 64 unique DEGs. We found approximately the same results (325 loops mapping to 64 unique genes) in the pear group. From these lists we extracted the loops with at least one anchor found at the promoter region of the DEGs which corresponds to thirty-five unique DEGs (Table 1, in apples). The transcription of these genes is likely regulated by the enhancer region we identified by HiChIP (opposite loop anchor in Table 1), and some are potentially involved in fat distribution heterogeneity (*HOXA*, *BDNF*, *IL33*, *EPHX2*, *IGF2BP1*).

In the last decade, large population studies have used GWAS to explore the genetic influences on WHR [48]. Historically, human GWAS studies have been performed without regard to chromatin structure. We next asked if the location of genes important to clinical phenotypes like WHR, or genes involved in adipocyte function, might overlap with our atlas of chromatin structure in ADSCs. We identified known SNPs associated with WHR (48 studies reporting 4797 unique SNPs annotated to genes listed in Supplementary Table S4) located inside chromatin loops: all loops, differential loops in apples (described in Figure 4B), and differential loops in pears (described in Supplementary Figure S3E). This is a conservative analysis as SNPs that were nearby, but not exactly inside the loop anchors, were not included. We found 417 WHR-associated SNPs in loop anchors identified in our study (in apple and in pear samples).

**Figure 5:**
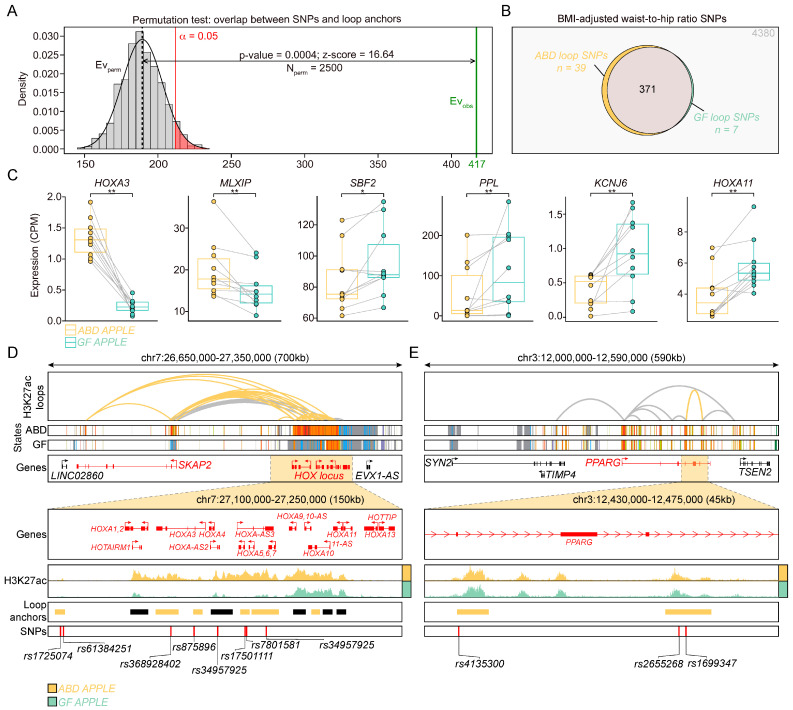
Integration of loop anchors and GWAS-SNPs associated with WHR. (**A**) Permutation test showing the overlap between loop anchors and SNPs. Green lane shows the observed overlap (n = 417) and the gray histogram shows the expected distribution of overlaps by shuffling the SNP positions 2500 times. Dotted line indicates the mean expected overlap, which was used to calculate significance at *p*-value < 0.05 (red lane). (**B**) Venn diagram showing number of overlapping SNPs with loop anchors, grouped by enriched loops in the ABD (yellow) or GF (green) samples and common loops between the ABD and GF samples (grey). (**C**) Boxplots showing the level of expression of genes annotated to loop anchors overlapping with WHR-SNP in ABD and GF-ADSCs. Paired Wilcoxon test * *p* < 0.05 ** *p* < 0.01 (**D**,**E**) Genome browser visualization of *SKAP2-HOXA* locus (**D**) and *PPARG* locus (**E**) in ABD (yellow) and in GF (green) samples. From top to bottom: H3K27ac loops, ChromHMM states, gene annotation. The zoom in windows of *HOXA* locus (**D**) and *PPARG* last exons (**E**) show H3K27ac in the ABD (yellow) and GF (green) samples, the loop anchors (colored in yellow when belonging to the ABD-enriched loop), and WHR-SNPs. Color coding for ChromHMM plots is the same as Figure 2C.

To ascertain if this could be a random finding, we performed a random permutation test and found that the number of SNPs that overlap with loop anchors was significantly higher than the number expected by random (Figure 5A).

From these 417 SNPs, 39 were found in ABD-enriched loops and 7 were found in GF-enriched loops (Figure 5B and Table 2). Some of the genes annotated to the depot-enriched loops, in other words having a WHR-SNP in their anchor, were also differentially expressed in one of the adipose tissue depots as depicted in the plots in Figure 5C that emphasize the differences in expression from each individual in both groups (HOXA3, MLXIP, SBF2, PPL, KCNJ6, HOXA11). Interestingly, MLXIP is a bHLH transcription factor that dimerizes with CHREB to regulate the expression of genes involved in glucose metabolism, glycogen synthesis, triglyceride synthesis, and insulin signaling [49]. MLXIP expression has also been implicated in the development of metabolic diseases such as obesity, insulin resistance, and T2DM [50,51]. Additionally, MLXIP has also been recently identified as a marker of a sub-population of human adipocytes that are highly responsive to insulin [52].

The overlap between WHR-SNPs and loop anchors identified as enriched in the ABD samples was more revealing than the seven WHR-SNPs found in the anchors of the GF-enriched loop library (Figure 5B). Indeed, nine SNPs were found in loop anchors in the HOXA cluster on chromosome 7 (Figure 5D) and three SNPs were found in loop anchors in the PPARG gene (Figure 5E). The HOXA genes are differentially regulated in ABD vs. GF adipose tissue, preadipocytes, and adipocytes [15,27,46], whereas PPAR gamma is a master transcription factor enriched in preadipocytes and adipocytes, necessary for adipogenesis and also regulates fat and glucose metabolism [53].

Taken together, these results suggest that these SNPs may affect WHR by the regulation of ABD but not GF adipose tissue function and that this effect is driven by differential looping in the ADSCs and potentially in mature adipocytes.

## 3. Discussion

Chromatin loops can link enhancers physically close to their target genes and help to better understand the alterations of gene transcription that affect disease. Our work, described here for the first time in primary human ADSCs, provides an extensive atlas of 3D-associated regulatory interactions. To gain insight into the potential function of these long-range chromatin interactions, we integrated the HiChIP interactome with the genes differentially expressed between the ABD and GF samples, with genes known to influence adiposity and cardiometabolic traits, and with GWAS-SNPs that are associated with WHR. We also established a list of loops that describe differential 3D genomic interactions in two groups of women (apple and pear-shaped). Importantly, some of these interactions were associated with ABD and/or GF-ADSC gene expression profiles that we highlighted by RNAPII ChIP-seq analysis performed in parallel.

In our earlier study where we were limited to using linear annotation, we showed that only a small fraction of body-shape-specific open chromatin regions were annotated to DEGs [46]. We proposed that long-range genomic interactions mediated by chromatin looping were likely involved in the differential gene expression patterns. Thus, in the present study, we used H3K27ac HiChIP to interrogate active enhancer-associated looping in regulating depot-enriched gene expression and this resulted in the identification of 35 unique DEGs with associated loop anchors (Table 1). The transcription of these genes is potentially regulated through the enhancer loop interaction revealed in our HiChIP data set (opposite loop anchor in Table 1) and some likely influence differential fat distribution (*HOXA*, *BDNF*, *IL33*, *EPHX2*, *IGF2BP1*). Importantly, our work discovered a potential new key transcription factor such as ZFP36L1, an inhibitor of adipogenesis [54,55], as a master regulator of depot-specific gene expression. We described 14 loops at its promoter (Table 1), reflecting its high potential of interaction with other distally located genomic regions. Other genes related to obesity and/or adipogenesis were identified by our HiChIP analysis as regulators of ABD vs. GF gene transcription, such as METTL15 [56] and RBM4 [57]. Overall, our study supports the idea that long-range chromatin loops may affect the development or differentiation of ADSCs and could explain in part subcutaneous adipose tissue dysfunction in diseases such as T2D or PCOS.

Previously reported large population studies have relied on GWAS to link genes to WHR [48]. Importantly, the gene connections have been performed relying largely on linear annotation and have not typically considered the importance of longer range chromatin interactions that are defined using more involved chromatin looping methods. Using our HiChIP data set, we connected SNPs known to be associated with WHR (48 studies reporting 4797 unique SNPs annotated to genes) with key chromatin loops: all loops (Figure 4B and Supplementary Figure S3E). It should be noted that this is a conservative estimate because we narrowly defined the SNPs to be located within the loop anchors and did not consider closely associated anchors in this analysis. Importantly, this revealed genes that were also differentially expressed in one of the adipose tissue depots (Figure 5C) that are known to influence adipose tissue function including *HOXA3*, *MLXIP*, *SBF2*, and *PPL*. Taken together, these findings demonstrate that genomic interactions play an important role in adipose depot-specific gene regulation in human ADSCs. In addition, by comparing the loops identified between the two adipose tissue depots studied (ABD vs. GF), we highlighted depot-enriched chromatin interactions that likely contribute to depot-selective 3D chromatin organization; this organization influences gene transcription and therefore the distinct functional phenotypes in ABD vs. GF-ADSCs.

We also report here for the first time in human primary ADSCs, that differential histone modifications at gene promoters influence patterns of depot-selective gene expression in ABD vs. GF depots. By studying the correlation between histone marks and differential gene expression between ABD and GF-ADSCs, our work revealed that combinations of histone marks are associated with transcriptional activity in ABD and GF-ADSCs. When the individual marks were combined to generate a combinatorial set of ChromHMM emission patterns, the data are even more supportive of the model.

However, we cannot formally conclude whether differential gene expression is the cause or consequence of differential histone modifications. Henikoff et al. showed that histone modifications were more likely the consequences than the causes of transcription, especially for H3K4me3 [58]. Regardless of the direction, these histone marks provide a stable memory of recent transcriptional activity and provide a template for a robust mechanism to sustain the observed differential pattern of transcription between depots.

A limitation of our work is that we used H3K27Ac HiChIP to identify the depot-specific connectome. However, a depot-enriched loop might be identified as specific due to the fact that those regions exhibit a high depot-enriched H3K27ac signal. We cannot conclude if it is this the result of an actual architectural change or simply a difference in H3K27ac at these anchors.

We focused here on loops associated with genes that were differentially expressed in ADSCs across different depots in apple vs. pear-shaped women. It should be noted that all other key genes involved in adipose function were not differentially expressed in our study. Taken together, these and prior experiments in human ADSCs reveal a potential epigenomic mechanism by which the differential growth and function of adipose tissue depots lead to common metabolic diseases.

## 4. Materials and Methods

### 4.1. Participants, Tissue Collection, and Isolation of Human Adipose-Derived Stem Cells

The method of recruitment, clinical, and biochemical parameters of subjects were previously published by Divoux A. et al. [46]. All procedures were performed under a research protocol approved by the AdventHealth Institutional Review Board. A subgroup of 10 healthy premenopausal, weight-stable women were used for this study. Five women displayed lower body adiposity characterized by a waist-to-hip ratio (WHR) < 0.78 (pear group; age = 34 ± 9.6 years; BMI = 29.2 ± 2.26 kg/m^2^) and five women displayed upper body adiposity, characterized by a WHR > 0.85 (apple group; age = 38 ± 8.1 years; BMI = 28.6 ± 3.54 kg/m^2^). Briefly, paired abdominal and gluteofemoral subcutaneous white adipose tissue samples were obtained from each participant and the stromal–vascular fractions (SVFs) were isolated by 45 min collagenase digestion (collagenase type I, Worthington). SVFs were plated and grown in proliferation medium containing 2.5% FBS, FGF, and EGF. Human adipose-derived stem cell (ADSC) populations were enriched as previously described [14]. The cells presenting at their surface the endothelial marker CD31 (MAB2148-C, MilliporeSigma, Burlington, MA, USA) were removed by magnetic beads.

### 4.2. Chromatin Immunoprecipitations

Chromatin immunoprecipitations (ChIPs) were performed on confluent ADSCs and analyzed as described [59]. ChIP grade Diagenode (Denville, NJ, USA) rabbit anti-H3K4me3 (C15410003), rabbit anti-H3K4me2 (pAb-035-050), rabbit anti-H3K27me3 (C15410069), and Abcam (Waltham, MA, USA) rabbit anti-H3K27Ac (ab4729) were used to study the histone marks. The CTCF antibody from Active Motif (Carlsbad, CA, USA) (61311) was used to study CTCF boundary sites. Rabbit anti-RNAPII (abcam, ab5095) was used to study the binding of the elongating form of RNA polymerase II (Serine 2 phospho form).

### 4.3. Assay for Transposase-Accessible Chromatin (ATAC)

ATAC was performed as previously described by Divoux A. et al. [15].

### 4.4. HiChIP Assay

Approximately 5 × 10^6^ cells were crosslinked in 1% formaldehyde (methanol-free, dissolved in phosphate-buffered saline—PBS) for 10 min at room temperature in a 10 mL final volume. Formaldehyde was quenched with the addition of 1.5 mL 1M glycine for 5 min at room temperature. Cells were scraped and lysed in lysis buffer (1% Triton x−100, 0.1% SDS, 150 mM NaCl, 1 mM EDTA, and 20 mM Tris, pH 8.0) for 1 h in rotation in a cold room. Isolated nuclei were pelleted by centrifugation, resuspended in 100 μL 0.5% SDS, and incubated for 10 min at 65 °C. SDS was quenched by the addition of Triton-X for 15 min at 37 °C. Nuclei were incubated overnight at 37 °C in a vigorous shaker (speed—850 rpm) in the presence of MboI (375U). The following day, the samples were incubated at 65 °C for 20 min to heat the inactivate MboI. Samples were left at room temperature for 20 min to cool down. Biotin fill-in of sticky ends was performed for 1 h at 37 °C in a vigorous shaker (speed—850 rpm) followed by ligation of blunt ends at room temperature for 6 h while rotating. Nuclei were spun, resuspended in lysis buffer in the presence of 5 μg H3K27ac antibody (ab4729, Abcam, Waltham, MA, USA), and incubated overnight on a rotator at 4 °C. The next day, antibody chromatin complexes were pulled down with protein A paramagnetic beads and sequentially washed: once in wash buffer 1 (1% Triton, 0.1% SDS, 150 mM NaCl, 1 mM EDTA, 20 mM Tris, pH 8.0, and 0.1% NaDOC), twice in wash buffer 2 (1% Triton, 0.1% SDS, 500 mM NaCl, 1 mM EDTA, 20 mM Tris, pH 8.0, and 0.1% NaDOC), once in wash buffer 3 (0.25 M LiCl, 0.5% NP−40, 1 mM EDTA, 20 mM Tris, pH 8.0, 0.5% NaDOC), and once in TE-buffer. After the removal of TE-buffer, DNA was eluted from the beads in an elution buffer. DNA was quantified with the Qubit dsDNA HS kit. Approximately, 40–50 ng DNA was used for biotin pull-down with streptavidin paramagnetic beads. Sequencing libraries were constructed with the Nugen Ovation Ultralow V2 kit (Tecan, Mannedorf, Switzerland) according to the manufacturer’s recommendations. Libraries were quantified with the Quibit dsDNA HS kit and subjected to bioanalyzer fragment analysis before paired-end sequencing.

### 4.5. HiChIP Data Processing

HiChIP data were analyzed using the default parameters of nf-core/hic (https://zenodo.org/records/2669513, accessed on 20 December 2023; version 1.3.0). In summary, the following steps were followed: (1) mapping to the *hg19* reference genome using a two-step strategy to rescue reads spanning the ligation sites (bowtie2) [60]; (2) detection for valid interaction products; (3) duplicates removal; and (4) generating raw and normalized contact maps using the ICE algorithm at various resolutions using a cooler. The quality control of the sample was included in the pipeline (*HiC-Pro* [61]). A/B compartments, saddle plots, and insulation scores were calculated using GENOVA [62]. Representative interaction heat maps were generated using cloops2 [63] with the *-corr* option. We used hichipper [64] to identify chromatin loops, using the consensus H3K27ac ChIP-seq peaks per group. Significant differential looping was calculated using diffloop [65] with *-nreplicates* set to 3 and *-nsamples* set to 8.

Loop anchor positions were overlapped with BMI-adjusted waist–hip ratio SNPs (EFO:0007788) and the permutation test was performed to test the significance of overlap compared to permutated (n = 2500) locations using the *regioneR* package [66].

### 4.6. Sequencing Library Preparation

RNA-seq, ATAC-seq, ChIP-seq, and HiChIP libraries were prepared and sequenced using standard Illumina protocols for a HiSeq 2500 instrument (Illumina, San Diego, CA, USA).

### 4.7. RNA Sequencing and Analysis

RNA sequencing was performed as described by Divoux [15]. The raw RNA-seq reads’ sequencing quality was evaluated by *FastQC* and the reads were aligned to the *hg19* reference genome using STAR (version 2.7.7a) [67]. Genes were quantified using *featureCounts* from Rsubread (version 2.4.0) [68]. The R package, edgeR with paired analysis, was used for differential gene expression analysis with cutoffs CPM > 3 in more than 4 samples. *p*-value < 0.05 was used to determine statistical significance for differentially expressed genes. DEGs were used for k-means clustering to create modules and visualized as a heat map.

### 4.8. ChIP-seq and ATAC-seq Analysis

Sequencing quality was evaluated by the *FastQC* software (v0.12.0). Reads were mapped to the human reference genome (*hg19*) using the default parameters of *BWA MEM* aligner [69]. Low mapping quality reads (MAPQ < 10), reads mapping to ENCODE human blacklisted regions [70], and duplicated reads were discarded from the downstream analyses, using *bedtools intersectBed* [71] and *samtools rmdup* [72]. Coverage profiles represent reads per kilobase million (RPKM) values, calculated using deeptools2 *bamCoverage* [73] and visualized in IGV.

### 4.9. Gene Set Enrichment and Visualization

EnrichR was used for gene set enrichment and visualization [74]. The enrichment was calculated to the hallmark gene set of the Molecular Signature Database (MSigDb). Pathways with *p*-values < 0.05 were selected as significant.

### 4.10. Chromatin State Discovery with ChromHMM

Tissue-specific chromatin states were identified using the ChromHMM (version 1.21) hidden Markov model (HMM) [43]. Bam files from RNAPII, CTCF, H3K27ac, H3K27me3, H3K4me2, H3K4me3 ChIP-seq, and ATAC-seq were binarized into default 200 bp bins using the function *BinarizeBam* from each of the 5 ABD-ADSC and 5 GF-ADSC samples in each group (apple and pear), as previously described [75]. We ran ChromHMM with a range of possible states and settled on a 10 states model as it accurately captured information from higher state models and provided sufficient resolution to identify biologically meaningful patterns in a reproducible way [43].

## Figures and Tables

**Table 1:** List of DEGs with at least one loop anchor located at their promoter.

DEG		LOOP					ANCHOR1					ANCHOR2				
DEG	Cluster	ID	Overlap	Width	Type	Sig	Annotation	Dist. from TSS	ENS_ID	Gene Name	Gene Type	Annotation	Dist. from TSS	ENS_ID	Gene Name	Gene Type
HOXA11	3	19124	anchor1	8628	e-p	down	promoter-TSS (NM_019102)	−94	ENSG00000106004	*HOXA5*	protein-coding	non-coding (NR_038832, exon 3 of 3)	4272	ENSG00000122592	*HOXA7*	protein-coding
HOXA11	3	19125	anchor1	23,154	e-p	down	promoter-TSS (NM_019102)	−94	ENSG00000106004	*HOXA5*	protein-coding	intron (NR_037940, intron 1 of 2)	−1392	ENSG00000078399	*HOXA9*	protein-coding
HOXA11	3	19127	anchor1	35,064	e-p	down	promoter-TSS (NM_019102)	−94	ENSG00000106004	*HOXA5*	protein-coding	intron (NR_037939, intron 1 of 1)	1021	ENSG00000253293	*HOXA10*	protein-coding
HOXA11	3	19126	anchor1	30,023	e-p	down	promoter-TSS (NM_019102)	−94	ENSG00000106004	*HOXA5*	protein-coding	exon (NM_018951, exon 1 of 2)	522	ENSG00000253293	*HOXA10*	protein-coding
GALNT16	3	35883	anchor1	12,524	e-p	ns	promoter-TSS (NM_004926)	−870	ENSG00000185650	*ZFP36L1*	protein-coding	Intergenic	−11,065	ENSG00000185650	*ZFP36L1*	protein-coding
GALNT16	3	35884	anchor1	20,626	e-p	ns	promoter-TSS (NM_004926)	−870	ENSG00000185650	*ZFP36L1*	protein-coding	Intergenic	−19,168	ENSG00000185650	*ZFP36L1*	protein-coding
GALNT16	3	35885	anchor1	47,834	e-p	ns	promoter-TSS (NM_004926)	−870	ENSG00000185650	*ZFP36L1*	protein-coding	Intergenic	−46,375	ENSG00000185650	*ZFP36L1*	protein-coding
ABHD14A-ACY1	3	10292	anchor1	12,344	e-p	ns	promoter-TSS (NM_004704)	150	ENSG00000041880	*PARP3*	protein-coding	promoter-TSS (NM_080865)	−523	ENSG00000180929	*GPR62*	protein-coding
ABHD14A-ACY1	3	10293	anchor1	24,976	e-p	ns	promoter-TSS (NM_004704)	150	ENSG00000041880	*PARP3*	protein-coding	promoter-TSS (NM_020418)	−24	ENSG00000090097	*PCBP4*	protein-coding
ABHD14A-ACY1	3	10294	anchor1	32,056	e-p	ns	promoter-TSS (NM_004704)	150	ENSG00000041880	*PARP3*	protein-coding	promoter-TSS (NM_032750)	61	ENSG00000114779	*ABHD14B*	protein-coding
ABHD14A-ACY1	3	10295	anchor1	42,148	e-p	ns	promoter-TSS (NM_004704)	150	ENSG00000041880	*PARP3*	protein-coding	intron (NM_001198898, intron 2 of 13)	1127	ENSG00000243989	*ACY1*	protein-coding
ABHD14A-ACY1	3	10296	anchor1	53,012	e-p	ns	promoter-TSS (NM_004704)	150	ENSG00000041880	*PARP3*	protein-coding	intron (NM_000992, intron 1 of 3)	369	ENSG00000162244	*RPL29*	protein-coding
ABHD14A-ACY1	3	10297	anchor1	112,696	e-p	ns	promoter-TSS (NM_004704)	150	ENSG00000041880	*PARP3*	protein-coding	intron (NM_001947, intron 1 of 2)	1362	ENSG00000164086	*DUSP7*	protein-coding
ABHD14A-ACY1	3	10298	anchor1	146,827	e-p	ns	promoter-TSS (NM_004704)	150	ENSG00000041880	*PARP3*	protein-coding	intron (NM_001161580, intron 9 of 9)	−24,228	NA	*LINC00696*	ncRNA
CFD	2	45670	anchor1	123,402	e-p	ns	intron (NM_001317335, intron 1 of 4)	760	ENSG00000197766	*CFD*	protein-coding	promoter-TSS (NM_024100)	−501	ENSG00000065268	*WDR18*	protein-coding
RIN1	2	29943	anchor1	70,293	e-p	ns	exon (NM_003793, exon 1 of 13)	193	ENSG00000174080	*CTSF*	protein-coding	promoter-TSS (NM_001198843)	4	ENSG00000173933	*RBM4*	protein-coding
PLEKHA4	2	48434	anchor1	26,441	e-p	ns	promoter-TSS (NR_130317)	−39	ENSG00000105467	*SYNGR4*	protein-coding	intron (NM_006801, intron 1 of 4)	782	ENSG00000105438	*KDELR1*	protein-coding
GORAB-AS1	2	3998	anchor1	99,172	e-p	ns	Intergenic	−31,865	NA	*GORAB-AS1*	ncRNA	promoter-TSS (NM_022716)	−485	ENSG00000116132	*PRRX1*	protein-coding
RAI14	5	13716	anchor1	28,634	e-p	ns	intron (NM_001145520, intron 1 of 17)	1670	ENSG00000039560	*RAI14*	protein-coding	promoter-TSS (NM_001145523)	−764	ENSG00000039560	*RAI14*	protein-coding
RAI14	5	13724	anchor1	257,285	e-p	ns	intron (NM_001145520, intron 1 of 17)	1670	ENSG00000039560	*RAI14*	protein-coding	promoter-TSS (NM_002853)	58	ENSG00000113456	*RAD1*	protein-coding
BDNF	5	28813	anchor1	409,349	e-p	ns	promoter-TSS (NM_170734)	−18	ENSG00000176697	*BDNF*	protein-coding	promoter-TSS (NM_031217)	646	ENSG00000169519	*METTL15*	protein-coding
HOXA9	5	19120	anchor1	9859	e-p	down	Intergenic	−3159	ENSG00000197576	*HOXA4*	protein-coding	promoter-TSS (NM_019102)	−94	ENSG00000106004	*HOXA5*	protein-coding
IL33	4	22820	anchor1	15,077	e-p	ns	promoter-TSS (NM_001314046)	29	ENSG00000137033	*IL33*	protein-coding	intron (NM_001199640, intron 1 of 6)	−6168	ENSG00000137033	*IL33*	protein-coding
CHMP1B-AS1	4	45102	anchor1	7276	e-p	ns	promoter-TSS (NM_020412)	−40	ENSG00000255112	*CHMP1B*	protein-coding	intron (NM_001261444, intron 1 of 7)	896	ENSG00000141404	*GNAL*	protein-coding
CHMP1B-AS1	4	45103	anchor1	96,022	e-p	ns	promoter-TSS (NM_020412)	−40	ENSG00000255112	*CHMP1B*	protein-coding	Intergenic	−34,112	ENSG00000141401	*IMPA2*	protein-coding
MIR210HG	4	28219	anchor1	7749	e-p	ns	promoter-TSS (NR_038262)	−231	ENSG00000247095	*MIR210HG*	ncRNA	promoter-TSS (NM_001286583)	−33	ENSG00000070047	*PHRF1*	protein-coding
HSD17B6	4	32706	anchor1	89,094	e-p	ns	promoter-TSS (NM_005419)	−96	ENSG00000170581	*STAT2*	protein-coding	TTS (NM_012064)	129	ENSG00000111602	*TIMELESS*	protein-coding
HSD17B6	4	32707	anchor1	102,550	e-p	ns	promoter-TSS (NM_005419)	−96	ENSG00000170581	*STAT2*	protein-coding	Intergenic	−5825	ENSG00000176422	*SPRYD4*	protein-coding
HSD17B6	4	32708	anchor1	108,572	e-p	ns	promoter-TSS (NM_005419)	−96	ENSG00000170581	*STAT2*	protein-coding	intron (NM_207344, intron 1 of 1)	197	ENSG00000176422	*SPRYD4*	protein-coding
COL7A1	6	10135	anchor1	6365	e-p	ns	promoter-TSS (NM_001317138)	−734	ENSG00000114268	*PFKFB4*	protein-coding	promoter-TSS (NM_033199)	−121	ENSG00000145040	*UCN2*	protein-coding
COL7A1	6	10136	anchor1	39,463	e-p	ns	promoter-TSS (NM_001317138)	−734	ENSG00000114268	*PFKFB4*	protein-coding	Intergenic	−1840	ENSG00000114270	*COL7A1*	protein-coding
COL7A1	6	10137	anchor1	77,961	e-p	ns	promoter-TSS (NM_001317138)	−734	ENSG00000114268	*PFKFB4*	protein-coding	promoter-TSS (NM_022911)	−37	ENSG00000225697	*SLC26A6*	protein-coding
PODNL1	6	47001	anchor1	21,746	e-p	ns	non-coding (NR_036515, exon 1 of 1)	2808	NA	*LOC284454*	ncRNA	promoter-TSS (NR_146095)	−180	ENSG00000187556	*NANOS3*	protein-coding
CPNE7	6	40745	anchor1	52,925	e-p	ns	Intergenic	−1189	ENSG00000197912	*SPG7*	protein-coding	promoter-TSS (NM_000977)	−533	ENSG00000167526	*RPL13*	protein-coding
HSD17B14	6	48432	anchor1	111,879	e-p	ns	non-coding (NR_130317, exon 6 of 6)	7398	ENSG00000142227	*EMP3*	protein-coding	promoter-TSS (NM_031485)	−706	ENSG00000105447	*GRWD1*	protein-coding
HSD17B14	6	48433	anchor1	135,056	e-p	ns	non-coding (NR_130317, exon 6 of 6)	7398	ENSG00000142227	*EMP3*	protein-coding	promoter-TSS (NM_004228)	−754	ENSG00000105443	*CYTH2*	protein-coding
HOXA11	3	19120	anchor2	9859	e-p	down	Intergenic	−3159	ENSG00000197576	*HOXA4*	protein-coding	promoter-TSS (NM_019102)	−94	ENSG00000106004	*HOXA5*	protein-coding
HOXA11	3	19110	anchor2	44,790	e-p	down	intron (NR_038367, intron 1 of 1)	2891	ENSG00000233429	*HOTAIRM1*	ncRNA	promoter-TSS (NM_019102)	−94	ENSG00000106004	*HOXA5*	protein-coding
HOXA11	3	19115	anchor2	32,933	e-p	down	intron (NM_153631, intron 2 of 3)	−8159	ENSG00000105996	*HOXA2*	protein-coding	promoter-TSS (NM_019102)	−94	ENSG00000106004	*HOXA5*	protein-coding
HOXA11	3	19099	anchor2	287,607	e-p	down	intron (NM_003930, intron 1 of 12)	1555	ENSG00000005020	*SKAP2*	protein-coding	promoter-TSS (NM_019102)	−94	ENSG00000106004	*HOXA5*	protein-coding
HOXA11	3	19092	anchor2	293,364	e-p	down	intron (NM_001303468, intron 3 of 12)	7312	ENSG00000005020	*SKAP2*	protein-coding	promoter-TSS (NM_019102)	−94	ENSG00000106004	*HOXA5*	protein-coding
GALNT16	3	35722	anchor2	852,295	e-p	ns	intron (NM_001321817, intron 8 of 11)	118,897	ENSG00000182185	*RAD51B*	protein-coding	promoter-TSS (NM_004926)	−870	ENSG00000185650	*ZFP36L1*	protein-coding
GALNT16	3	35737	anchor2	653,186	e-p	ns	intron (NM_001321817, intron 8 of 11)	318,006	ENSG00000182185	*RAD51B*	protein-coding	promoter-TSS (NM_004926)	−870	ENSG00000185650	*ZFP36L1*	protein-coding
GALNT16	3	35749	anchor2	546,374	e-p	ns	intron (NM_001321817, intron 8 of 11)	380,377	NA	*LOC100996664*	ncRNA	promoter-TSS (NM_004926)	−870	ENSG00000185650	*ZFP36L1*	protein-coding
GALNT16	3	35779	anchor2	329,101	e-p	ns	intron (NM_001321817, intron 10 of 11)	163,104	NA	*LOC100996664*	ncRNA	promoter-TSS (NM_004926)	−870	ENSG00000185650	*ZFP36L1*	protein-coding
GALNT16	3	35797	anchor2	285,117	e-p	ns	intron (NM_001321818, intron 10 of 10)	119,120	NA	*LOC100996664*	ncRNA	promoter-TSS (NM_004926)	−870	ENSG00000185650	*ZFP36L1*	protein-coding
GALNT16	3	35821	anchor2	248,544	e-p	ns	intron (NM_001321818, intron 10 of 10)	82,547	NA	*LOC100996664*	ncRNA	promoter-TSS (NM_004926)	−870	ENSG00000185650	*ZFP36L1*	protein-coding
GALNT16	3	35841	anchor2	156,840	e-p	ns	intron (NM_001321818, intron 10 of 10)	−9157	NA	*LOC100996664*	ncRNA	promoter-TSS (NM_004926)	−870	ENSG00000185650	*ZFP36L1*	protein-coding
GALNT16	3	35850	anchor2	125,930	e-p	ns	intron (NM_001321818, intron 10 of 10)	−40,067	NA	*LOC100996664*	ncRNA	promoter-TSS (NM_004926)	−870	ENSG00000185650	*ZFP36L1*	protein-coding
GALNT16	3	35855	anchor2	111,357	e-p	ns	TTS (NM_001321818)	−54,640	NA	*LOC100996664*	ncRNA	promoter-TSS (NM_004926)	−870	ENSG00000185650	*ZFP36L1*	protein-coding
GALNT16	3	35861	anchor2	99,498	e-p	ns	Intergenic	−66,499	NA	*LOC100996664*	ncRNA	promoter-TSS (NM_004926)	−870	ENSG00000185650	*ZFP36L1*	protein-coding
GALNT16	3	35868	anchor2	90,796	e-p	ns	Intergenic	−75,201	NA	*LOC100996664*	ncRNA	promoter-TSS (NM_004926)	−870	ENSG00000185650	*ZFP36L1*	protein-coding
GALNT16	3	35874	anchor2	77,461	e-p	ns	Intergenic	76,591	ENSG00000185650	*ZFP36L1*	protein-coding	promoter-TSS (NM_004926)	−870	ENSG00000185650	*ZFP36L1*	protein-coding
GALNT16	3	35878	anchor2	39,487	e-p	ns	Intergenic	38,617	ENSG00000185650	*ZFP36L1*	protein-coding	promoter-TSS (NM_004926)	−870	ENSG00000185650	*ZFP36L1*	protein-coding
GALNT16	3	35881	anchor2	8795	e-p	ns	Intergenic	7925	ENSG00000185650	*ZFP36L1*	protein-coding	promoter-TSS (NM_004926)	−870	ENSG00000185650	*ZFP36L1*	protein-coding
EGFL8	3	16744	anchor2	82,077	e-p	ns	Intergenic	−4749	ENSG00000168477	*TNXB*	protein-coding	promoter-TSS (NM_022107).6	−641	ENSG00000213654	*GPSM3*	protein-coding
EGFL8	3	16752	anchor2	67,171	e-p	ns	promoter-TSS (NM_001136153).2	−747	ENSG00000213676	*ATF6B*	protein-coding	promoter-TSS (NM_022107).6	−641	ENSG00000213654	*GPSM3*	protein-coding
EGFL8	3	16756	anchor2	43,230	e-p	ns	promoter-TSS (NM_030651)	149	ENSG00000204314	*PRRT1*	protein-coding	promoter-TSS (NM_022107).6	−641	ENSG00000213654	*GPSM3*	protein-coding
EGFL8	3	16759	anchor2	18,816	e-p	ns	promoter-TSS (NM_001371437)	−2	NA	*NA*	NA	promoter-TSS (NM_022107).6	−641	ENSG00000213654	*GPSM3*	protein-coding
EGFL8	3	16760	anchor2	6385	e-p	ns	TTS (NM_022107).6	423	ENSG00000204304	*PBX2*	protein-coding	promoter-TSS (NM_022107).6	−641	ENSG00000213654	*GPSM3*	protein-coding
SELENBP1	3	3317	anchor2	72,559	e-p	ns	promoter-TSS (NM_001330721)	−25	ENSG00000143393	*PI4KB*	protein-coding	exon (NM_002796, exon 2 of 7)	427	ENSG00000159377	*PSMB4*	protein-coding
RAP1GAP	2	960	anchor2	63,962	e-p	ns	promoter-TSS (NM_001113347)	−389	ENSG00000117298	*ECE1*	protein-coding	intron (NM_001113348, intron 1 of 18)	1538	ENSG00000117298	*ECE1*	protein-coding
EPHX2	2	21394	anchor2	21,110	e-p	ns	promoter-TSS (NM_001831)	−968	ENSG00000120885	*CLU*	protein-coding	intron (NM_182826, intron 1 of 5)	2718	ENSG00000168077	*SCARA3*	protein-coding
PLEKHG4	2	39878	anchor2	83,771	e-p	ns	promoter-TSS (NM_003789)	297	ENSG00000135722	*FBXL8*	protein-coding	intron (NM_001318202, intron 1 of 23)	3455	ENSG00000135723	*FHOD1*	protein-coding
TBXA2R	2	46172	anchor2	33,942	e-p	ns	intron (NM_006339, intron 1 of 9)	159	ENSG00000064961	*HMG20B*	protein-coding	promoter-TSS (NR_038865)	−171	ENSG00000006638	*TBXA2R*	protein-coding
TBXA2R	2	46175	anchor2	27,033	e-p	ns	TTS (NM_006339)	−5466	ENSG00000179855	*GIPC3*	protein-coding	promoter-TSS (NR_038865)	−171	ENSG00000006638	*TBXA2R*	protein-coding
SULT1A3	2	39468	anchor2	64,396	e-p	ns	promoter-TSS (NM_001040056)	7	ENSG00000102882	*MAPK3*	protein-coding	intron (NM_001193333, intron 7 of 11)	−1075	NA	*LOC606724*	pseudo
CA12	2	37448	anchor2	39,130	e-e	ns	promoter-TSS (NR_147233)	−956	NA	*TPM1-AS*	ncRNA	Intergenic	−32,387	ENSG00000103642	*LACTB*	protein-coding
GORAB-AS1	2	3978	anchor2	32,309	e-p	ns	promoter-TSS (NM_001320252)	−1	ENSG00000120370	*GORAB*	protein-coding	Intergenic	−31,865	NA	*GORAB-AS1*	ncRNA
TES	4	20235	anchor2	44,811	e-p	ns	promoter-TSS (NM_001172897)	−615	ENSG00000105974	*CAV1*	protein-coding	intron (NR_120506, intron 4 of 4)	44,196	ENSG00000105974	*CAV1*	protein-coding
MIR210HG	4	28217	anchor2	32,639	e-p	ns	promoter-TSS (NM_176795)	−473	ENSG00000174775	*HRAS*	protein-coding	promoter-TSS (NR_038262)	−231	ENSG00000247095	*MIR210HG*	ncRNA
HSD17B6	4	32695	anchor2	137,481	e-p	ns	promoter-TSS (NR_040053)	−752	ENSG00000181852	*RNF41*	protein-coding	promoter-TSS (NM_005419)	−96	ENSG00000170581	*STAT2*	protein-coding
HSD17B6	4	32702	anchor2	60,102	e-p	ns	intron (NM_004077, intron 1 of 10)	229	ENSG00000062485	*CS*	protein-coding	promoter-TSS (NM_005419)	−96	ENSG00000170581	*STAT2*	protein-coding
HSD17B6	4	32704	anchor2	44,090	e-p	ns	promoter-TSS (NM_014255)	−125	ENSG00000257727	*CNPY2*	protein-coding	promoter-TSS (NM_005419)	−96	ENSG00000170581	*STAT2*	protein-coding
COL7A1	6	10130	anchor2	87,150	e-p	ns	TTS (NM_001271022)	190	ENSG00000213689	*TREX1*	protein-coding	promoter-TSS (NM_001317138)	−734	ENSG00000114268	*PFKFB4*	protein-coding
PODNL1	6	46954	anchor2	109,067	e-p	ns	promoter-TSS (NM_001320561)	−709	ENSG00000104957	*CCDC130*	protein-coding	non-coding (NR_036515, exon 1 of 1)	2808	NA	*LOC284454*	ncRNA
PODNL1	6	46970	anchor2	65,696	e-p	ns	promoter-TSS (NM_014047)	−42	ENSG00000104979	*C19orf53*	protein-coding	non-coding (NR_036515, exon 1 of 1)	2808	NA	*LOC284454*	ncRNA
CPNE7	6	40657	anchor2	186,088	e-e	ns	promoter-TSS (NM_001242885)	−22	NA	*LOC100287036*	protein-coding	Intergenic	−1189	ENSG00000197912	*SPG7*	protein-coding
CPNE7	6	40720	anchor2	76,223	e-e	ns	promoter-TSS (NR_110931)	57	NA	*LOC101927817*	ncRNA	Intergenic	−1189	ENSG00000197912	*SPG7*	protein-coding
CPNE7	6	40622	anchor2	290,001	e-p	ns	promoter-TSS (NM_182531)	−506	ENSG00000170100	*ZNF778*	protein-coding	Intergenic	−1189	ENSG00000197912	*SPG7*	protein-coding
SPAAR	6	23267	anchor2	79,338	e-p	ns	promoter-TSS (NM_016446)	468	ENSG00000204930	*FAM221B*	protein-coding	TTS (NM_001039792)	1400	ENSG00000196196	*HRCT1*	protein-coding
IGF2BP1	6	43400	anchor2	51,386	e-p	ns	promoter-TSS (NR_038458)	−99	ENSG00000229980	*TOB1-AS1*	ncRNA	Intergenic	−49,988	ENSG00000141232	*TOB1*	protein-coding
HSD17B14	6	48426	anchor2	62,105	e-e	ns	promoter-TSS (NM_001331098)	5	ENSG00000178150	*ZNF114*	protein-coding	non-coding (NR_130317, exon 6 of 6)	7398	ENSG00000142227	*EMP3*	protein-coding
TSPAN13	6	19005	anchor2	64,574	e-p	ns	promoter-TSS (NM_020319)	285	ENSG00000136261	*BZW2*	protein-coding	Intergenic	−42,715	ENSG00000106537	*TSPAN13*	protein-coding

ns = not significant; NA = not applicable.

**Table 2:** List of WHR-SNPs overlapping with loop anchors found in both subcutaneous adipose tissue depots.

Unspecific Anchor		ABD-Specific Anchor		GF-Specific Anchor	
SNP_ID	Gene Name	SNP_ID	Gene Name	SNP_ID	Gene Name
rs12138127	*ZMIZ1-AS1*	rs1037925	*ARNTL2*	rs7907173	*LASTR*
rs7530102	*REEP2*	rs61955587	*B3GNT4*	rs2734741	*PPL*
rs3119837	*NA-BARX1*	rs1139653	*DNAJA3*	rs2957674	*SBF2*
rs3747636	*MIR3188-RPL39P38*	rs2074553	*DOT1L*	rs2853986	*RNU6-283P-FGFR3P1*
rs12078075	*GDF5*	rs2240128	*DOT1L*	rs273507	*MAST3*
rs762705	*DYRK1A-KCNJ6*	rs2835810	*DYRK1A-KCNJ6*	rs7350438	*LASTR*
rs758801	*PPL*	rs4722669	*GORAB-PRRX1*	rs2923125	*AMPD3*
rs12495674	*RAI1*	rs114709597	*H6PD-SPSB1*		
rs11724804	*RAI1*	rs564101206	*H6PD-SPSB1*		
rs55962025	*KANSL1*	rs10248288	*HOTTIP-EVX1-AS*		
rs2137234	*GATA4*	rs7801581	*HOXA11*		
rs9742	*SLC44A4*	rs17501111	*HOXA11*		
rs77881454	*C2*	rs9770544	*HOXA11-AS-HOXA13*		
rs6546164	*RNU6-682P-RPL10P19*	rs1725074	*HOXA2-HOXA3*		
rs34312154	*SMIM20-LINC02357*	rs61384251	*HOXA3*		
rs3782086	*PSORS1C1*	rs10827226	*NRP1*		
rs117737783	*DNM2*	rs875896	*HOXA7-HOXA9*		
rs12580347	*LOXL1*	rs34957925	*HOXA9, HOXA10*		
rs2277339	*RFLNA*	rs368928402	*HOXA-AS3, HOXA3*		
rs771846	*PHGR1*	rs8043060	*IQCH-AS1, IQCH*		
rs10827226	*NRP1*	rs28768122	*KMT5A*		
rs6480914	*HLA-DMB*	rs10514889	*MAPT*		
rs12419064	*LIN52*	rs9896485	*MAPT*		
rs982085		rs885683	*MAST3*		
rs34000	*PBRM1*	rs2048498	*MLXIP-LRRC43*		
rs3904600	*MLXIP*	rs925460	*MLXIP-LRRC43*		
rs4722669	*GORAB-PRRX1*	rs711082	*NAV3*		
rs56285369	*LY75, CD302*	rs2474724	*NRP1*		
rs9402211	*FLRT1, MACROD1*	rs4646342	*PEMT*		
rs7823561	*RPL35P2-NUDT3*	rs771846	*PHGR1*		
rs71511927	*MICB-DT*	rs750619494	*ABHD2*		
rs6994124	*MRPS18A-VEGFA*	rs747616512	*ABHD2*		
rs112875651	*RPS10-NUDT3*	rs4135300	*PPARG-TSEN2*		
rs2725308	*MIR9-1HG*	rs2655268	*PPARG-TSEN2*		
rs2166365	*LINC01142, LINC01681*	rs1699347	*PPARG-TSEN2*		
rs7256111	*MICB-DT*	rs778984966	*SMAD3*		
rs143384	*GDF5*	rs12140013	*THEMIS2*		
rs11664106	*SMCHD1-EMILIN2*	rs1583969	*ZFAT*		
rs2058914	*USP3*	rs55650389	*ZFAT*		
rs876476	*CLEC16A*				
rs2925979	*CMIP*				
rs12435046	*RAD51B*				
rs12042959	*SDCCAG8*				
rs780159	*ZMIZ1*				
rs7907173	*LASTR*				
rs793456	*COL8A1-CMSS1*				
rs797486	*DLEU1, DLEU7*				
rs8043060	*IQCH-AS1, IQCH*				
rs8071778	*CDK5RAP3-COPZ2*				
rs1139653	*DNAJA3*				
rs12575252	*TRIM66*				
rs12828016	*WNK1*				
rs3736485	*DMXL2*				
rs4646342	*PEMT*				
rs4660808	*PPIEL*				
rs6021889	*LINC01524*				
rs1122080					
rs459193	*RPL26P19-C5orf67*				
rs605203	*EHMT2-AS1*				
rs2276824	*PBRM1*				
rs2845885	*FLRT1, MACROD1*				
rs3810068	*SMCHD1-EMILIN2*				
rs7801581	*HOXA11*				
rs3741378	*SIPA1*				
rs3747577	*CORO7-PAM16, CORO7*				
rs1051921	*MLXIPL*				
rs544668	*TSPAN9*				
rs11893688	*ADAM17*				
rs2595004	*ATG7*				
rs807067	*PAQR7*				
rs380654	*COL24A1*				
rs7783857	*KLF14-H4P1*				
rs12868881	*NA-LINC02337*				
rs2957658	*AMPD3*				
rs6694768	*TRIM33*				
rs7025089	*MED27*				
rs11694173	*THADA*				
rs2747399	*TSHZ2*				
rs4871958	*EBF2*				
rs2835810	*DYRK1A-KCNJ6*				
rs2734741	*PPL*				
rs7166081	*SMAD3-AAGAB*				
rs4575098	*ADAMTS4*				
rs465002	*C5orf67*				
rs75766425	*NID2*				
rs9379082	*RREB1*				
rs79823890	*NID2*				
rs740746	*NHLRC2-ADRB1*				
rs750619494	*PLIN1*				
rs2284178	*HCP5*				
rs2921097	*PRAG1-RN7SL178P*				
rs2921036	*PRAG1-RN7SL178P*				
rs35190619	*NA-RN7SL178P*				
rs56367294	*MFHAS1*				
rs10098531	*RNU6-682P-RPL10P19*				
rs2797963	*KRT18P9-CYCSP55*				
rs10248288	*HOTTIP-EVX1-AS*				
rs57340203	*RREB1*				
rs3857546	*H1-4-H2BC5*				
rs11435482	*H3C9P-BTN3A2*				
rs9379850	*H3C9P-BTN3A2*				
rs4282054	*NT5DC2*				
rs7695004	*RBPJ*				
rs11697492	*LINC01524*				
rs532086	*C2*				
rs4646404	*PEMT*				
rs7224739	*RAI1*				
rs11649804	*RAI1*				
rs10514889	*MAPT*				
rs11653367	*KANSL1*				
rs377346776	*EYA1*				
rs7928917	*PNPLA2*				
rs4841580	*LINC00208-GATA4*				
rs10503426	*GATA4*				
rs2957674	*SBF2*				
rs12419342	*RAPSN*				
rs778984966	*SMAD3*				
rs76748772	*PEPD*				
rs1264376	*HCG20-LINC00243*				
rs2535324	*HCG20*				
rs2853986	*RNU6-283P-FGFR3P1*				
rs7629072	*WDR82*				
rs885910	*DDR1*				
rs1265158	*POU5F1*				
rs2263314	*MICA*				
rs28436034	*MICA*				
rs730213	*LINC02875-TBX4*				
rs494620	*SLC44A4*				
rs521977	*SLC44A4*				
rs2844452	*C2*				
rs2734313	*TNXB*				
rs2856451	*TNXB*				
rs1150754	*TNXB*				
rs448231	*RNU6-682P-RPL10P19*				
rs6917995	*H3C9P-BTN3A2*				
rs17643342	*RNU6-682P-RPL10P19*				
rs313736	*COL24A1*				
rs804281	*GATA4*				
rs7826055	*GATA4*				
rs1228024	*NUP160-PTPRJ*				
rs6501784	*GRB2*				
rs11386443	*FNDC3B*				
rs3773842	*DLG1*				
rs4690196	*DGKQ*				
rs11724232	*NA-LINC01365*				
rs1567651	*SMIM20-LINC02357*				
rs5025813	*SMIM20-LINC02357*				
rs14323	*H1-10-AS1*, *H1-10*				
rs6764238	*H1-10-AS1-RPL32P3*				
rs3131014	*CCHCR1*				
rs254431	*SPRY4-AS1*				
rs3095304	*PSORS1C1*				
rs77169818	*GALR1*				
rs2074553	*DOT1L*				
rs55818584	*DNMT1, S1PR2*				
rs55660036	*DNM2*				
rs273507	*MAST3*				
rs7246274	*PDE4C*				
rs11130362	*TKT*				
rs6068216	*LINC01524*				
rs28710106	*TSHZ2*				
rs62206548	*TSHZ2*				
rs6494407	*USP3*				
rs12441130	*LOXL1*				
rs7191812	*CORO7-PAM16*, *CORO7*				
rs1291695	*CORO7*, *CORO7-PAM16*, *VASN*				
rs4785960	*CORO7-PAM16*, *CORO7*				
rs116734066	*DNAJC27-AS1-EFR3B*				
rs79761284	*LINC01381-DNMT3A*				
rs17745484	*DNMT3A*				
rs7954697	*SCARB1*				
rs61953572	*DNAH10*, *CCDC92, DNAH10OS*				
rs752843328	*RFLNA*				
rs825508	*RFLNA*				
rs1906937	*RFLNA*				
rs35777573	*PHGR1*				
rs1473781	*RPAP1*				
rs201612157	*OR7E159P-GNG2*				
rs117311385	*GNG2*				
rs28469812	*RILPL2*				
rs117209788	*RILPL1*				
rs137963709	*RILPL1-MIR3908*				
rs148118721	*ATP6V0A2*				
rs6488898	*ATP6V0A2*				
rs2271049	*HIP1R*				
rs940904	*PITPNM2*				
rs139192229	*DNAH10OS*, *DNAH10, CCDC92*				
rs59364353	*RFLNA*				
rs17753769	*PPP1R14BP5-CENPW*				
rs1725074	*HOXA2-HOXA3*				
rs368928402	*HOXA-AS3*, *HOXA3*				
rs875896	*HOXA7-HOXA9*				
rs34957925	*HOXA9*, *HOXA10*				
rs17501111	*HOXA11*				
rs9770544	*HOXA11-AS-HOXA13*				
rs28576490	*JAZF1*				
rs57291069	*NKX2-6-NA*				
rs144362803	*TRIB1-NA*				
rs1583969	*ZFAT*				
rs7834111	*ZFAT*				
rs2474724	*NRP1*				
rs35727416	*EYA1*				
rs35416759	*RILPL2*				
rs181981038	*BAZ1B*				
rs7487608	*MLXIP*				
rs11057291	*MLXIP*				
rs2048498	*MLXIP-LRRC43*				
rs61955587	*B3GNT4*				
rs117269855	*KNTC1-HCAR2*				
rs2277346	*KNTC1*				
rs3121911	*LINC01681*				
rs1332952	*LINC01681*, *LINC01142*				
rs12131969	*HAUS4P1-GORAB-AS1*				
rs11808978	*GORAB-PRRX1*				
rs2641431	*SMG6*				
rs8081548	*POLR2A-Y_RNA*				
rs11641142	*CMIP*				
rs114709597	*H6PD-SPSB1*				
rs564101206	*H6PD-SPSB1*				
rs2999140	*ASAP3-E2F2*				
rs140681455	*FUBP1*				
rs2927327	*CMIP*				
rs62064595	*RNA5SP443-ARHGAP27*				
rs9303523	*MAPT*				
rs8080903	*MAPT*				
rs720856	*RAI1*				
rs3818717	*RAI1*				
rs36058389	*ALKBH5-LLGL1*				
rs2240128	*DOT1L*				
rs8191979	*SHC1*				
rs147847496	*DPM3-HMGN2P18*				
rs756916254					
rs35444446					
rs201632637	*KLF14-H4P1*				
rs2309651	*AFF3*				
rs56186131	*LY75*, *LY75-CD302*				
rs145272880	*PLA2R1*				
rs7594266	*GRB14-COBLL1*				
rs148358468	*TTLL4*				
rs4135300	*PPARG-TSEN2*				
rs11213979	*SIK2*				
rs60906625	*SSPN*				
rs61914547	*SSPN-ITPR2*				
rs1037925	*ARNTL2*				
rs144737537	*SP7-SP1*				
rs12426763	*CISTR-RN7SKP289*				
rs4332564	*HOXC13-HOXC12*				
rs2366149	*HOXC13-HOXC12*				
rs75493807	*HOXC6*, *HOXC9*, *HOXC-AS2*				
rs10778496	*RFX4*				
rs1922432	*RFX4*				
rs157512	*C5orf67*				
rs10070929	*FGF1*, *SPRY4-AS1*				
rs9379081	*RREB1*				
rs1620540	*GNG2*				
rs730566	*TMA7-ATRIP*				
rs34365302	*DNAH1*				
rs2655268	*PPARG-TSEN2*				
rs1699347	*PPARG-TSEN2*				
rs67409736	*STAB1*				
rs11176017	*RPL21P18-RNA5SP362*				
rs716446	*RFX4*				
rs925460	*MLXIP-LRRC43*				
rs7316114	*CLIP1-ZCCHC8*				
rs140323250	*NA-MIR148A*				
rs287621	*KLF14-H4P1*				
rs854793	*MYO15A*				
rs9896485	*MAPT*				
rs4135268	*PPARG*				
rs12358916	*ARID5B-RTKN2*				
rs4290124	*ARID5B-RTKN2*				
rs7917772	*SFXN2*				
rs2244524	*SFXN2*				
rs11199755	*NA-RPL19P16*				
rs61876729	*GATD1-CEND1*				
rs7107271	*GATD1-CEND1*				
rs12799550	*MACROD1*, *FLRT1*				
rs1006207	*MACROD1*, *FLRT1*				
rs2186643	*MACROD1*, *FLRT1*				
rs17158803	*FLRT1*, *MACROD1*				
rs73502335	*PRDX5-CCDC88B*				
rs1662185	*PRDX5-CCDC88B*				
rs55869750	*AHNAK*				
rs67308910	*EML3*				
rs1893458	*INTS5-C11orf98*				
rs7978072	*RASSF8-BHLHE41*				
rs77757339	*BHLHE41*, *SSPN*				
rs7955859	*SSPN*				
rs7134738	*SSPN*				
rs9668178	*SSPN*				
rs3094014	*HCP5*				
rs2596473	*LINC01149-HCP5*				
rs9380180	*SUCLA2P1-RANP1*				
rs2797964	*KRT18P9-CYCSP55*				
rs1759637	*RPL35P2-NUDT3*				
rs12195665	*MICB-DT*				
rs10661543	*MICB-DT*				
rs2534681	*MICB*				
rs62395355	*MICB*				
rs12204413	*MRPS18A-VEGFA*				
rs145416558	*FAM13A*				
rs2905757	*HCG22*				
rs116594542	*RPS10-NUDT3*				
rs2763977	*HSPA1A*				
rs2607015	*VARS1*				
rs10223666	*VEGFA-LINC02537*				
rs35208023	*MIR9-1HG*				
rs34469991	*PC*				
rs55650389	*ZFAT*				
rs144831544	*NCR3-UQCRHP1*				
rs2857694	*AIF1-PRRC2A*				
rs2763981	*SLC44A4,*				
rs644774	*SLC44A4*				
rs9267653	*SLC44A4*				
rs7301643	*NA-HOXC13-AS*				
rs67330701	*MYEOV*				
rs10750786	*BRD9P1*				
rs313734	*COL24A1*				
rs12734458	*COL24A1*				
rs2990657	*LINC01142*, *LINC01681*				
rs71455259	*HOXC13-AS*				
rs10784510	*LINC02425*				
rs711082	*NAV3*				
rs7139153	*NA-HOXC13-AS*				
rs7307887	*KNTC1-HCAR2*				
rs7896335	*NA-RPL19P16*				
rs2509985	*AHNAK*				
rs34341044	*PBRM1*				
rs6772089	*IL17RD*				
rs111593386	*GLYCTK-AS1-DNAH1*				
rs62265318	*EFCC1*				
rs41264253	*PBXIP1*				
rs60925903	*EFR3B*				
rs11124930	*THADA*				
rs12466434	*LINC01937-TWIST2*				
rs852425	*ACTB*				
rs17145717	*BAZ1B*				
rs143214539	*PPP1R14BP5-CENPW*				
rs9381248	*MRPS18A-VEGFA*				
rs28768122	*KMT5A*				
rs4759364	*KNTC1-HCAR2*				
rs80024005	*VPS37B-ABCB9*				
rs111854458	*CCDC92*				
rs2378280	*ZC3H11B-SLC30A10*				
rs73078824	*PBRM1*				
rs4786485	*VASN*, *CORO7*, *PAM16*				
rs73507245	*PAM16*, *CORO7-PAM16*				
rs60570301	*ELL*				
rs1363120	*PGPEP1-GDF15*				
rs885683	*MAST3*				
rs72832896	*RNA5SP443-ARHGAP27*				
rs112881773	*EMILIN2*				
rs4378729	*MIR3188-RPL39P38*				
rs11670016	*RPL39P38-LSM4*				
rs61876744	*PNPLA2*				
rs2008019	*EBPL*				
rs13412	*P3H4*				
rs854788	*MYO15A*				
rs7219992	*ZBTB4*, *SLC35G6*				
rs7218457	*LINC02210-CRHR1*				
rs55762977	*SLC25A19-GRB2*				
rs550600266	*TRMT11*				
rs73243890	*LINC02357*				
rs421215	*LINC01948*				
rs61384251	*HOXA3*				
rs2108864	*FGF1-LINC01844*				
rs73005768	*ESR1*				
rs811458	*ASTN2*				
rs7350438	*LASTR*				
rs144100226	*KRT18P9-CYCSP55*				
rs2923125	*AMPD3*				
rs60521849	*KANSL1*				
rs650180	*TSPAN9*				
rs57561811	*SLC38A6-PRKCH*				
rs28378811	*LINC00316-MTCO1P3*				
rs4371408	*LINC01524*				
rs10992447	*BICD2*				
rs2246618	*MICB-PPIAP9*				
rs2904597	*MICB-DT*				
rs2844498	*MICB*				
rs3130277	*FKBPL-PRRT1*				
rs77318243	*HLA-DMB*				
rs3132584	*TUBB*				
rs1264375	*HCG20-LINC00243*				
rs1076829	*DHX16*				
rs2857595	*NCR3-UQCRHP1*				
rs3132450	*PRRC2A*				
rs28752890	*LINC02571-HLA-B*				
rs2844495	*MICB-PPIAP9*				
rs11057401	*CCDC92*				
rs6931262	*RREB1*				
rs150999300	*LINC02775-LINC01348*				
rs12140013	*THEMIS2*				
rs190930640	*THSD4*				
rs769422497	*FAM168A*				
rs565732042	*LIN52*				
rs199913532	*KIDINS220*				
rs1982963	*NID2*				
rs17223632	*SPRY4-AS1, FGF1*				
rs747616512	*PLIN1*				
rs370499275	*PLIN1*				
rs12549058	*EYA1*				
rs11989744	*NKX2-6-NA*				
rs16996700	*LINC01524*				
rs532552327	*RSPRY1*				
rs222487	*COX7A2L*				
rs139516594					
rs7975017	*SSPN*				
rs2590838	*PBRM1*				
rs1894633	*DNM3*				
rs10783615	*HOXC12*				
rs12489828	*NT5DC2*				
rs1872992	*SSPN-ITPR2*				
rs13241538	*KLF14-H4P1*				
rs10743579	*SSPN-ITPR2*				
rs12443634	*CMIP*				
rs6088552	*PIGU*				

## Data Availability

All sequencing data have been deposited to the NCBI GEO database (http://www.ncbi.nlm.nih.gov/geo/, accessed on 20 December 2023) under accession number GSE#176603, GSE#224770, GSE#224777.

## Supplementary Materials

The following supporting information can be downloaded at: https://www.mdpi.com/article/10.3390/ijms25010437/s1.

## References

[1] Canoy D., Boekholdt S.M., Wareham N., Luben R., Welch A., Bingham S., Buchan I., Day N., Khaw K.T. (2007). Body fat distribution and risk of coronary heart disease in men and women in the European Prospective Investigation Into Cancer and Nutrition in Norfolk cohort: A population-based prospective study. Circulation.

[2] Santosa S., Jensen M.D. (2008). Why are we shaped differently, and why does it matter?. Am. J. Physiol. Endocrinol. Metab..

[3] Karpe F., Pinnick K.E. (2015). Biology of upper-body and lower-body adipose tissue—Link to whole-body phenotypes. Nat. Rev. Endocrinol..

[4] Rask-Andersen M., Karlsson T., Ek W.E., Johansson Å. (2019). Genome-wide association study of body fat distribution identifies adiposity loci and sex-specific genetic effects. Nat. Commun..

[5] Jia Q., Morgan-Bathke M.E., Jensen M.D. (2020). Adipose tissue macrophage burden, systemic inflammation, and insulin resistance. Am. J. Physiol. Endocrinol. Metab..

[6] Patel P., Abate N. (2013). Body fat distribution and insulin resistance. Nutrients.

[7] Karastergiou K., Bredella M.A., Lee M.J., Smith S.R., Fried S.K., Miller K.K. (2016). Growth hormone receptor expression in human gluteal versus abdominal subcutaneous adipose tissue: Association with body shape. Obesity.

[8] Lee M.J., Pramyothin P., Karastergiou K., Fried S.K. (2014). Deconstructing the roles of glucocorticoids in adipose tissue biology and the development of central obesity. Biochim. Biophys. Acta.

[9] Newell-Fugate A.E. (2017). The role of sex steroids in white adipose tissue adipocyte function. Reproduction.

[10] Kuo F.C., Neville M.J., Sabaratnam R., Wesolowska-Andersen A., Phillips D., Wittemans L.B.L., van Dam A.D., Loh N.Y., Todorčević M., Denton N. (2022). HOTAIR interacts with PRC2 complex regulating the regional preadipocyte transcriptome and human fat distribution. Cell Rep..

[11] Denton N., Pinnick K.E., Karpe F. (2018). Cartilage oligomeric matrix protein is differentially expressed in human subcutaneous adipose tissue and regulates adipogenesis. Mol. Metab..

[12] Loh N.Y., Minchin J.E.N., Pinnick K.E., Verma M., Todorčević M., Denton N., Moustafa J.E., Kemp J.P., Gregson C.L., Evans D.M. (2020). RSPO3 impacts body fat distribution and regulates adipose cell biology in vitro. Nat. Commun..

[13] Pinnick K.E., Nicholson G., Manolopoulos K.N., McQuaid S.E., Valet P., Frayn K.N., Denton N., Min J.L., Zondervan K.T., Fleckner J. (2014). Distinct developmental profile of lower-body adipose tissue defines resistance against obesity-associated metabolic complications. Diabetes.

[14] Divoux A., Eroshkin A., Erdos E., Sandor K., Osborne T.F., Smith S.R. (2021). DNA Methylation as a Marker of Body Shape in Premenopausal Women. Front. Genet..

[15] Divoux A., Sandor K., Bojcsuk D., Talukder A., Li X., Balint B.L., Osborne T.F., Smith S.R. (2018). Differential open chromatin profile and transcriptomic signature define depot-specific human subcutaneous preadipocytes: Primary outcomes. Clin. Epigenetics.

[16] Bonev B., Cavalli G. (2016). Organization and function of the 3D genome. Nat. Rev. Genet..

[17] Su J.H., Zheng P., Kinrot S.S., Bintu B., Zhuang X. (2020). Genome-Scale Imaging of the 3D Organization and Transcriptional Activity of Chromatin. Cell.

[18] Belton J.M., McCord R.P., Gibcus J.H., Naumova N., Zhan Y., Dekker J. (2012). Hi-C: A comprehensive technique to capture the conformation of genomes. Methods.

[19] Eagen K.P. (2018). Principles of Chromosome Architecture Revealed by Hi-C. Trends Biochem. Sci..

[20] Lafontaine D.L., Yang L., Dekker J., Gibcus J.H. (2021). Hi-C 3.0: Improved Protocol for Genome-Wide Chromosome Conformation Capture. Curr. Protoc..

[21] Mumbach M.R., Rubin A.J., Flynn R.A., Dai C., Khavari P.A., Greenleaf W.J., Chang H.Y. (2016). HiChIP: Efficient and sensitive analysis of protein-directed genome architecture. Nat. Methods.

[22] Cordero A.D., Callihan E.C., Said R., Alowais Y., Paffhausen E.S., Bracht J.R. (2020). Epigenetic Regulation of Neuregulin-1 Tunes White Adipose Stem Cell Differentiation. Cells.

[23] Bagchi D.P., MacDougald O.A. (2021). Wnt Signaling: From Mesenchymal Cell Fate to Lipogenesis and Other Mature Adipocyte Functions. Diabetes.

[24] Yang Loureiro Z., Joyce S., DeSouza T., Solivan-Rivera J., Desai A., Skritakis P., Yang Q., Ziegler R., Zhong D., Nguyen T.T. (2023). Wnt signaling preserves progenitor cell multipotency during adipose tissue development. Nat. Metab..

[25] Khan T., Muise E.S., Iyengar P., Wang Z.V., Chandalia M., Abate N., Zhang B.B., Bonaldo P., Chua S., Scherer P.E. (2009). Metabolic dysregulation and adipose tissue fibrosis: Role of collagen VI. Mol. Cell. Biol..

[26] Spencer M., Unal R., Zhu B., Rasouli N., McGehee R.E., Peterson C.A., Kern P.A. (2011). Adipose tissue extracellular matrix and vascular abnormalities in obesity and insulin resistance. J. Clin. Endocrinol. Metab..

[27] Karastergiou K., Fried S.K., Xie H., Lee M.J., Divoux A., Rosencrantz M.A., Chang R.J., Smith S.R. (2013). Distinct developmental signatures of human abdominal and gluteal subcutaneous adipose tissue depots. J. Clin. Endocrinol. Metab..

[28] Dutta A.B., Lank D.S., Przanowska R.K., Przanowski P., Wang L., Nguyen B., Walavalkar N.M., Duarte F.M., Guertin M.J. (2023). Kinetic networks identify TWIST2 as a key regulatory node in adipogenesis. Genome Res..

[29] Fisk H.L., Childs C.E., Miles E.A., Ayres R., Noakes P.S., Paras-Chavez C., Antoun E., Lillycrop K.A., Calder P.C. (2022). Dysregulation of Subcutaneous White Adipose Tissue Inflammatory Environment Modelling in Non-Insulin Resistant Obesity and Responses to Omega-3 Fatty Acids—A Double Blind, Randomised Clinical Trial. Front. Immunol..

[30] Frances L., Tavernier G., Viguerie N. (2021). Adipose-Derived Lipid-Binding Proteins: The Good, the Bad and the Metabolic Diseases. Int. J. Mol. Sci..

[31] Perugini J., Bordoni L., Venema W., Acciarini S., Cinti S., Gabbianelli R., Giordano A. (2019). Zic1 mRNA is transiently upregulated in subcutaneous fat of acutely cold-exposed mice. J. Cell. Physiol..

[32] Gallini R., Lindblom P., Bondjers C., Betsholtz C., Andrae J. (2016). PDGF-A and PDGF-B induces cardiac fibrosis in transgenic mice. Exp. Cell Res..

[33] Ostendorf T., Boor P., van Roeyen C.R., Floege J. (2014). Platelet-derived growth factors (PDGFs) in glomerular and tubulointerstitial fibrosis. Kidney Int. Suppl..

[34] Chen X., Zhao C., Xu Y., Huang K., Wang Y., Wang X., Zhou X., Pang W., Yang G., Yu T. (2021). Adipose-specific BMP and activin membrane-bound inhibitor (BAMBI) deletion promotes adipogenesis by accelerating ROS production. J. Biol. Chem..

[35] Araujo N., Sledziona J., Noothi S.K., Burikhanov R., Hebbar N., Ganguly S., Shrestha-Bhattarai T., Zhu B., Katz W.S., Zhang Y. (2022). Tumor Suppressor Par-4 Regulates Complement Factor C3 and Obesity. Front. Oncol..

[36] Shi X., Shao X., Liu B., Lv M., Pandey P., Guo C., Zhang R., Zhang Y. (2020). Genome-wide screening of functional long noncoding RNAs in the epicardial adipose tissues of atrial fibrillation. Biochim. Biophys. Acta Mol. Basis Dis..

[37] Passaro A., Miselli M.A., Sanz J.M., Dalla Nora E., Morieri M.L., Colonna R., Pišot R., Zuliani G. (2017). Gene expression regional differences in human subcutaneous adipose tissue. BMC Genom..

[38] Guo T., Gupta A., Yu J., Granados J.Z., Gandhi A.Y., Evers B.M., Iyengar P., Infante R.E. (2021). LIFR-α-dependent adipocyte signaling in obesity limits adipose expansion contributing to fatty liver disease. iScience.

[39] Jo D., Son Y., Yoon G., Song J., Kim O.Y. (2020). Role of Adiponectin and Brain Derived Neurotrophic Factor in Metabolic Regulation Involved in Adiposity and Body Fat Browning. J. Clin. Med..

[40] Svensson P.A., Wahlstrand B., Olsson M., Froguel P., Falchi M., Bergman R.N., McTernan P.G., Hedner T., Carlsson L.M., Jacobson P. (2014). CDKN2B expression and subcutaneous adipose tissue expandability: Possible influence of the 9p21 atherosclerosis locus. Biochem. Biophys. Res. Commun..

[41] Zaragosi L.E., Wdziekonski B., Villageois P., Keophiphath M., Maumus M., Tchkonia T., Bourlier V., Mohsen-Kanson T., Ladoux A., Elabd C. (2010). Activin a plays a critical role in proliferation and differentiation of human adipose progenitors. Diabetes.

[42] Anatskaya O.V., Runov A.L., Ponomartsev S.V., Vonsky M.S., Elmuratov A.U., Vinogradov A.E. (2023). Long-Term Transcriptomic Changes and Cardiomyocyte Hyperpolyploidy after Lactose Intolerance in Neonatal Rats. Int. J. Mol. Sci..

[43] Ernst J., Kellis M. (2017). Chromatin-state discovery and genome annotation with ChromHMM. Nat. Protoc..

[44] Gozzi G., Chelbi S.T., Manni P., Alberti L., Fonda S., Saponaro S., Fabbiani L., Rivasi F., Benhattar J., Losi L. (2016). Promoter methylation and downregulated expression of the TBX15 gene in ovarian carcinoma. Oncol. Lett..

[45] Paço A., de Bessa Garcia S.A., Freitas R. (2020). Methylation in HOX Clusters and Its Applications in Cancer Therapy. Cells.

[46] Divoux A., Sandor K., Bojcsuk D., Yi F., Hopf M.E., Smith J.S., Balint B.L., Osborne T.F., Smith S.R. (2020). Fat Distribution in Women Is Associated With Depot-Specific Transcriptomic Signatures and Chromatin Structure. J. Endocr. Soc..

[47] Pan D.Z., Miao Z., Comenho C., Rajkumar S., Koka A., Lee S.H.T., Alvarez M., Kaminska D., Ko A., Sinsheimer J.S. (2021). Identification of TBX15 as an adipose master trans regulator of abdominal obesity genes. Genome Med..

[48] Huang L.O., Rauch A., Mazzaferro E., Preuss M., Carobbio S., Bayrak C.S., Chami N., Wang Z., Schick U.M., Yang N. (2021). Genome-wide discovery of genetic loci that uncouple excess adiposity from its comorbidities. Nat. Metab..

[49] Ahn B., Wan S., Jaiswal N., Vega R.B., Ayer D.E., Titchenell P.M., Han X., Won K.J., Kelly D.P. (2019). MondoA drives muscle lipid accumulation and insulin resistance. JCI Insight.

[50] Ahn B. (2023). The Function of MondoA and ChREBP Nutrient-Sensing Factors in Metabolic Disease. Int. J. Mol. Sci..

[51] Ke H., Luan Y., Wu S., Zhu Y., Tong X. (2021). The Role of Mondo Family Transcription Factors in Nutrient-Sensing and Obesity. Front. Endocrinol..

[52] Bäckdahl J., Franzén L., Massier L., Li Q., Jalkanen J., Gao H., Andersson A., Bhalla N., Thorell A., Rydén M. (2021). Spatial mapping reveals human adipocyte subpopulations with distinct sensitivities to insulin. Cell Metab..

[53] Tontonoz P., Spiegelman B.M. (2008). Fat and beyond: The diverse biology of PPARgamma. Annu. Rev. Biochem..

[54] Lin N.Y., Lin T.Y., Yang W.H., Wang S.C., Wang K.T., Su Y.L., Jiang Y.W., Chang G.D., Chang C.J. (2012). Differential expression and functional analysis of the tristetraprolin family during early differentiation of 3T3-L1 preadipocytes. Int. J. Biol. Sci..

[55] Tseng K.Y., Chen Y.H., Lin S. (2017). Zinc finger protein ZFP36L1 promotes osteoblastic differentiation but represses adipogenic differentiation of mouse multipotent cells. Oncotarget.

[56] Bradfield J.P., Vogelezang S., Felix J.F., Chesi A., Helgeland Ø., Horikoshi M., Karhunen V., Lowry E., Cousminer D.L., Ahluwalia T.S. (2019). A trans-ancestral meta-analysis of genome-wide association studies reveals loci associated with childhood obesity. Hum. Mol. Genet..

[57] Zhang P., Wu W., Ma C., Du C., Huang Y., Xu H., Li C., Cheng X., Hao R., Xu Y. (2022). RNA-Binding Proteins in the Regulation of Adipogenesis and Adipose Function. Cells.

[58] Henikoff S., Shilatifard A. (2011). Histone modification: Cause or cog?. Trends Genet. TIG.

[59] Daniel B., Balint B.L., Nagy Z.S., Nagy L. (2014). Mapping the genomic binding sites of the activated retinoid X receptor in murine bone marrow-derived macrophages using chromatin immunoprecipitation sequencing. Methods Mol. Biol..

[60] Langmead B., Salzberg S.L. (2012). Fast gapped-read alignment with Bowtie 2. Nat. Methods.

[61] Servant N., Varoquaux N., Lajoie B.R., Viara E., Chen C.J., Vert J.P., Heard E., Dekker J., Barillot E. (2015). HiC-Pro: An optimized and flexible pipeline for Hi-C data processing. Genome Biol..

[62] van der Weide R.H., van den Brand T., Haarhuis J.H.I., Teunissen H., Rowland B.D., de Wit E. (2021). Hi-C analyses with GENOVA: A case study with cohesin variants. NAR Genom. Bioinform..

[63] Cao Y., Liu S., Ren G., Tang Q., Zhao K. (2022). cLoops2: A full-stack comprehensive analytical tool for chromatin interactions. Nucleic Acids Res..

[64] Lareau C.A., Aryee M.J. (2018). hichipper: A preprocessing pipeline for calling DNA loops from HiChIP data. Nat. Methods.

[65] Lareau C.A., Aryee M.J. (2018). diffloop: A computational framework for identifying and analyzing differential DNA loops from sequencing data. Bioinformatics.

[66] Gel B., Díez-Villanueva A., Serra E., Buschbeck M., Peinado M.A., Malinverni R. (2016). regioneR: An R/Bioconductor package for the association analysis of genomic regions based on permutation tests. Bioinformatics.

[67] Dobin A., Davis C.A., Schlesinger F., Drenkow J., Zaleski C., Jha S., Batut P., Chaisson M., Gingeras T.R. (2013). STAR: Ultrafast universal RNA-seq aligner. Bioinformatics.

[68] Liao Y., Smyth G.K., Shi W. (2019). The R package Rsubread is easier, faster, cheaper and better for alignment and quantification of RNA sequencing reads. Nucleic Acids Res..

[69] Li H., Durbin R. (2010). Fast and accurate long-read alignment with Burrows-Wheeler transform. Bioinformatics.

[70] Amemiya H.M., Kundaje A., Boyle A.P. (2019). The ENCODE Blacklist: Identification of Problematic Regions of the Genome. Sci. Rep..

[71] Quinlan A.R., Hall I.M. (2010). BEDTools: A flexible suite of utilities for comparing genomic features. Bioinformatics.

[72] Li H., Handsaker B., Wysoker A., Fennell T., Ruan J., Homer N., Marth G., Abecasis G., Durbin R. (2009). The Sequence Alignment/Map format and SAMtools. Bioinformatics.

[73] Ramírez F., Ryan D.P., Grüning B., Bhardwaj V., Kilpert F., Richter A.S., Heyne S., Dündar F., Manke T. (2016). deepTools2: A next generation web server for deep-sequencing data analysis. Nucleic Acids Res..

[74] Chen E.Y., Tan C.M., Kou Y., Duan Q., Wang Z., Meirelles G.V., Clark N.R., Ma’ayan A. (2013). Enrichr: Interactive and collaborative HTML5 gene list enrichment analysis tool. BMC Bioinform..

[75] Ernst J., Kellis M. (2012). ChromHMM: Automating chromatin-state discovery and characterization. Nat. Methods.

