# Supplementary material for: An Atlas of Promoter Chromatin Modifications and HiChIP Regulatory Interactions in Human Subcutaneous Adipose-Derived Stem Cells
Source: Int J Mol Sci. 2023 Dec 28;25(1):437. doi: 10.3390/ijms25010437 (PMC10778978; doi:10.3390/ijms25010437)
Supplement: Supplementary file 1 [file ijms-25-00437-s001.zip › ijms-2705948-supplementary.pdf]

Supporting Information

Clinical parameters	Apple subjects (n=5)	Pear subjects (n=5)	p-value (Mann-Whitney test)
Age (Years)	38 ±7.3	34 ±8.6	0.40
Race(C/H/AA/other)	4/0/0/1	1/2/2/0	
**Adiposity Markers**			
BMI (kg/m²)	28.6 ±3.2	29.2 ±2.0	0.69
Weight (kg)	77.6 ±6.4	78.6 ±8.4	0.89
Total Fat mass (kg)	35.5 ±6.80	31.3 ±5.62	0.42
Total Lean mass (kg)	39.3 ±2.23	45.7 ±4.40	0.03
Fat Mass (%)	47 ±5.4	40 ±4.4	0.09
Lean Mass (%)	53 ±5.4	60 ±4.4	0.09
Leg FM/Total FM	0.34 ±0.05	0.47 ±0.05	0.01
Android/Gynoid FM	0.57 ±0.12	0.29 ±0.06	0.01
Visceral FM (g)	922 ±321	356 ±272	0.03
Liver Fat (%)	0.03 ±0.02	0.01 ±0.01	0.22
Waist to hip ratio	0.89 ±0.03	0.72 ±0.03	0.0079
**Metabolic Markers**			
Fasting Glucose (mg/dL)	85 ±5.06	88 ±4.43	0.44
Fasting Insulin (μ[iU]/mL)	6.54 ±3.10	7.06 ±3.69	0.84
HOMA-IR	1.4 ±0.7	1.5 ±0.8	0.84
HgbA1c (%)	5.36 ±0.17	5.30 ±0.38	0.90
HDL (mg/dL)	61.4 ±19.7	65.4 ±6.22	0.80
TGL (mg/dL)	87.2 ±31.1	57.0 ±14.7	0.21
Cholesterol (mg/dL)	185 ±40.5	173 ±23.6	0.84
TSH (mU/L)	2.06 ±0.80	1.48 ±0.32	0.22

Supplemental Table S1: Clinical and metabolic characteristics of the 10 women. C: Caucasian, H: Hispanic, AA: African American, FM: Fat Mass

Supplemental Figure S1

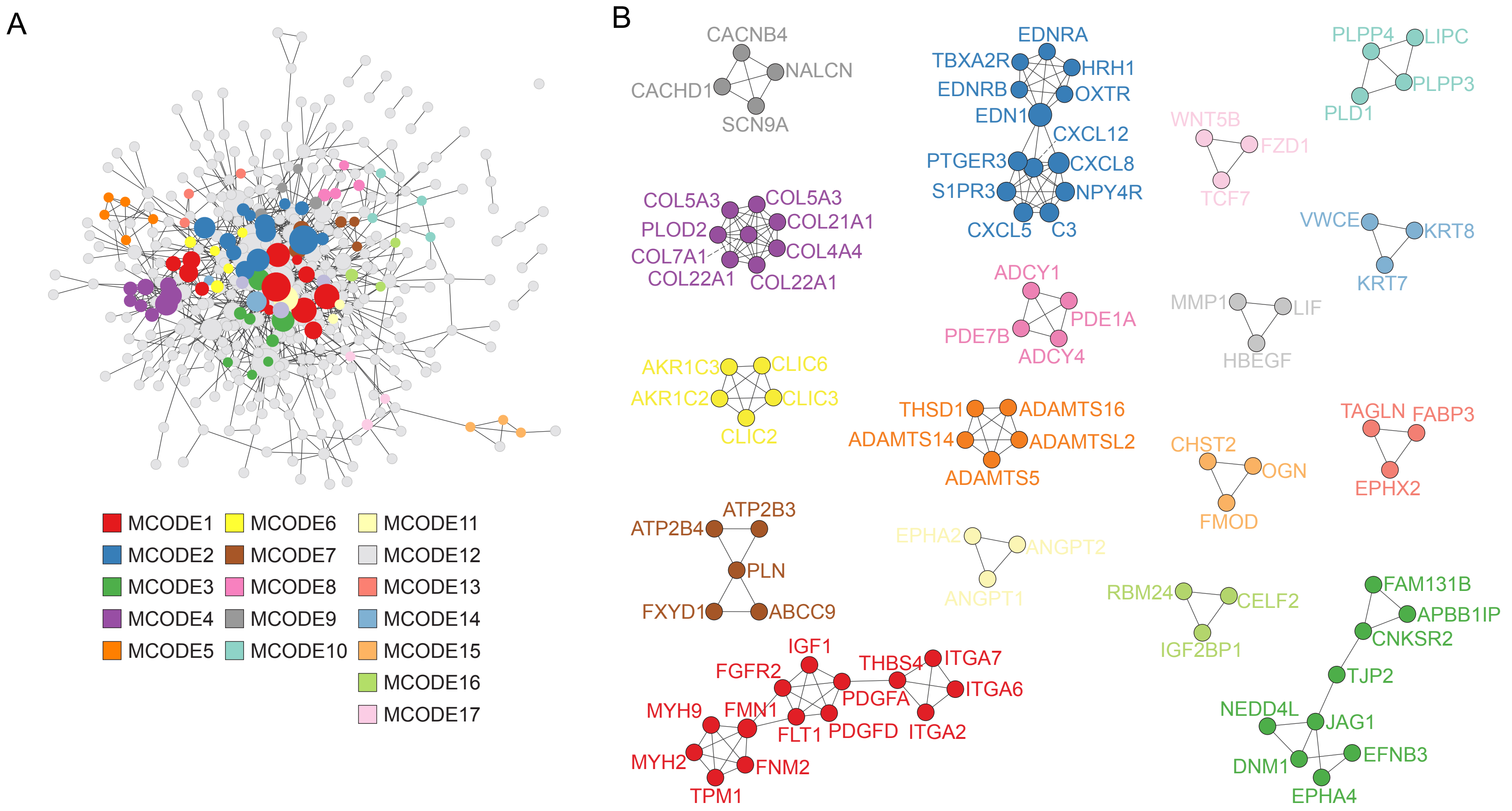

Figure S1: Protein-protein interaction network (A) and MCODE components (B) were identified in the DEG list (599 genes) using Metascape. The network and MCODE components were constructed on the base of physical interactions taken from the String server.

Supplemental Figure S2

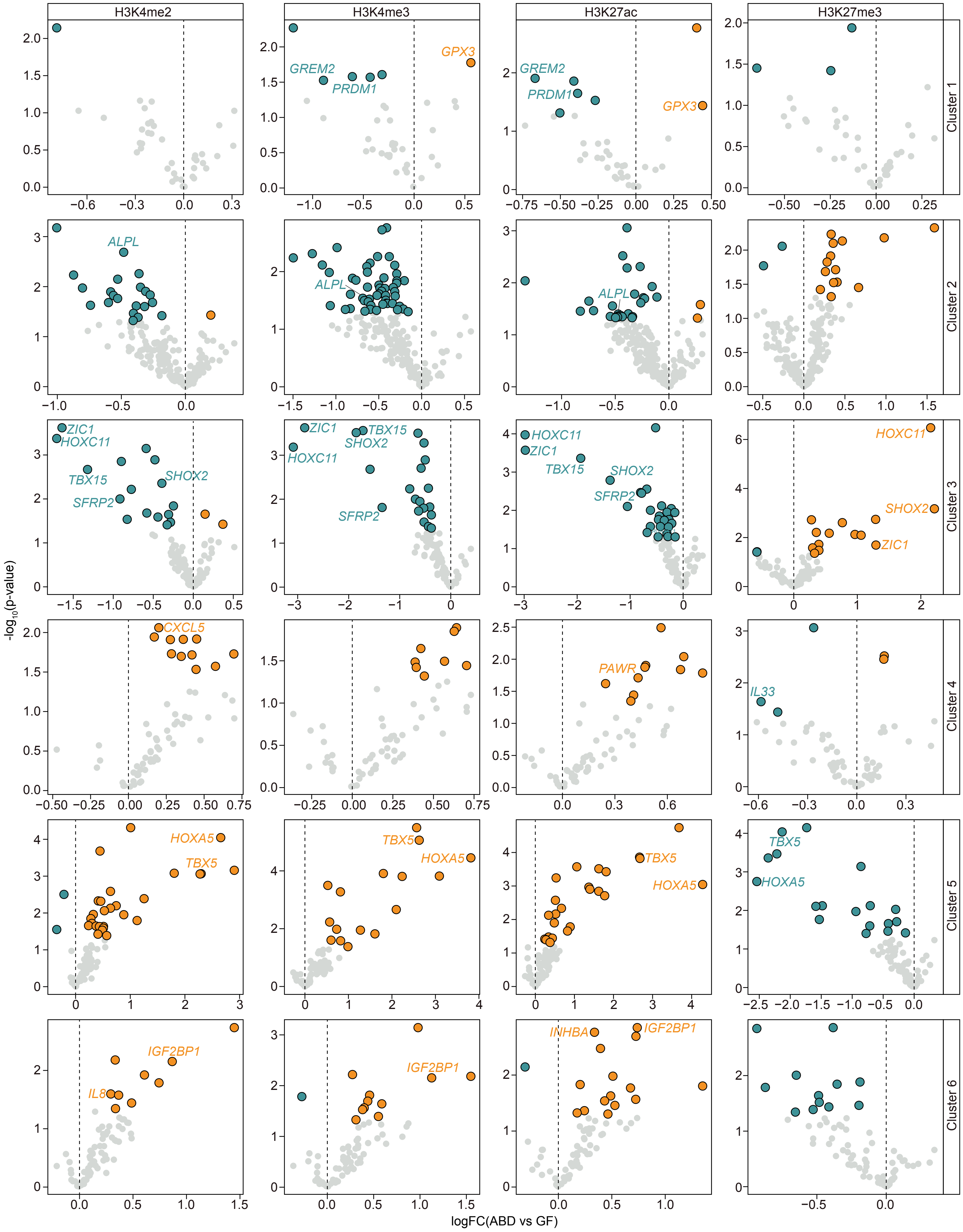

Figure S2: Association between depot-enriched expression and depot-enriched chromatin marks at the TSS (±2kbp) in pear samples. Volcano plots show for each genes and each histone marks studied the average fold change of the ChIP-seq signal between ABD and GF-ADSCs at the TSS. Data are representated by cluster of DEGs (rows). Negative fold changes (green) indicate chIP-seq signal significantly enriched in GF samples, positive fold changes (orange) indicate ChIP-seq signal significantly enriched in ABD samples.

Supplemental Figure S3

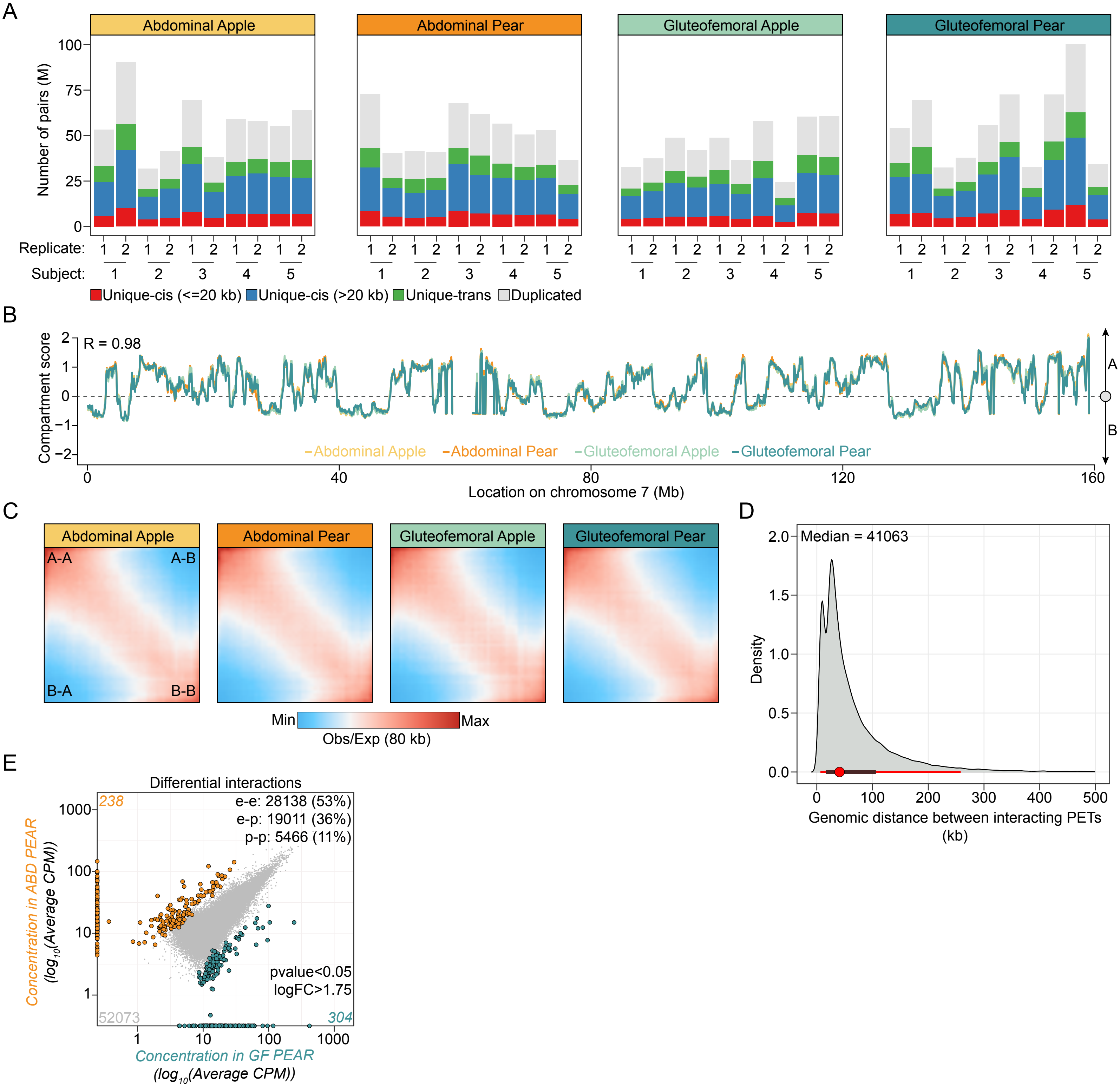

Figure S3: H3K27ac HiChIP descriptive statistics. (A) HiC-Pro contact statistics for all samples. Bar plots showing the uniquely mapped cis-short ranged contacts (≤20kb, red), uniquely mapped cis-long ranged contacts (>20kb, blue), uniquely mapped interchromosomal interactions (trans, green) and duplicated (gray). (B) Line plot showing the A/B compartmentalization score (first eigenvector) for chromosome 7 for each depot and body shape groups. (C) Saddle plots showing the genome-wide correlation of A/B compartments for each depot and body shape groups. (D) Density plot showing the size distribution of significant interactions. Median of 41,063 bp is indicated by a red dot. (E) Dot plot showing the correlation of read densities between apple ABD- and GF-ADSCs in pear subjects.
